# Comprehensive multi-cohort transcriptional meta-analysis of muscle diseases identifies a signature of disease severity

**DOI:** 10.1038/s41598-022-15003-1

**Published:** 2022-07-04

**Authors:** C. J. Walsh, J. Batt, M. S. Herridge, S. Mathur, G. D. Bader, P. Hu, P. Khatri, C. C. dos Santos

**Affiliations:** 1 Keenan Research Center for Biomedical Science, Saint Michael’s Hospital, Toronto, ON Canada; 2 Institute of Medical Sciences, University of Toronto, Toronto, ON Canada; 3 Interdepartmental Division of Critical Care, University of Toronto, Toronto, ON Canada; 4 Interdepartmental Division of Critical Care, University Health Network, University of Toronto, Toronto, ON Canada; 5 Department of Physical Therapy, University of Toronto, Toronto, ON Canada; 6 The Donnelly Center, University of Toronto, Toronto, ON Canada; 7 Department of Biochemistry and Medical Genetics, University of Manitoba, Winnipeg, MB Canada; 8 Stanford Institute for Immunity, Transplantation and Infection (ITI), Stanford University School of Medicine, Stanford, CA USA; 9 Department of Medicine, Stanford Center for Biomedical Informatics Research (BMIR), Stanford University, Stanford, CA USA

**Keywords:** Computational biology and bioinformatics, Data integration, Microarrays, Predictive medicine, Statistical methods, Diagnostic markers

## Abstract

Muscle diseases share common pathological features suggesting common underlying mechanisms. We hypothesized there is a common set of genes dysregulated across muscle diseases compared to healthy muscle and that these genes correlate with severity of muscle disease. We performed meta-analysis of transcriptional profiles of muscle biopsies from human muscle diseases and healthy controls. Studies obtained from public microarray repositories fulfilling quality criteria were divided into six categories: (i) immobility, (ii) inflammatory myopathies, (iii) intensive care unit (ICU) acquired weakness (ICUAW), (iv) congenital muscle diseases, (v) chronic systemic diseases, (vi) motor neuron disease. Patient cohorts were separated in discovery and validation cohorts retaining roughly equal proportions of samples for the disease categories. To remove bias towards a specific muscle disease category we repeated the meta-analysis five times by removing data sets corresponding to one muscle disease class at a time in a “leave-one-disease-out” analysis. We used 636 muscle tissue samples from 30 independent cohorts to identify a 52 gene signature (36 up-regulated and 16 down-regulated genes). We validated the discriminatory power of this signature in 657 muscle biopsies from 12 additional patient cohorts encompassing five categories of muscle diseases with an area under the receiver operating characteristic curve of 0.91, 83% sensitivity, and 85.3% specificity. The expression score of the gene signature inversely correlated with quadriceps muscle mass (r = −0.50, p-value = 0.011) in ICUAW and shoulder abduction strength (r = −0.77, p-value = 0.014) in amyotrophic lateral sclerosis (ALS). The signature also positively correlated with histologic assessment of muscle atrophy in ALS (r = 0.88, p-value = 1.62 × 10^–3^) and fibrosis in muscular dystrophy (Jonckheere trend test p-value = 4.45 × 10^–9^). Our results identify a conserved transcriptional signature associated with clinical and histologic muscle disease severity. Several genes in this conserved signature have not been previously associated with muscle disease severity.

## Introduction

Skeletal muscle diseases result in decreased muscle mass and muscle dysfunction thereby inducing physical disability and increased mortality^1^. Skeletal muscle dysfunction has been shown to contribute to decreased quality of life, increased disease morbidity and mortality in respiratory illness including for example, chronic obstructive pulmonary disease (COPD), pulmonary arterial hypertension and acute respiratory distress syndrome (ARDS)^2–4^. Despite the profound clinical implications, knowledge of the molecular mechanisms of muscle dysfunction, as well as objective, non-volitional methods to quantify the degree of muscle dysfunction are insufficient^5,6^. Understanding the pathomolecular mechanisms conserved across muscle diseases may provide vital insight to help develop therapies to ameliorate them.

A growing number of studies of human muscle disease have identified dysregulated gene expression that is associated with disease severity^1,7–9^. These studies however, are usually limited by relatively small sample sizes without external validation from independent cohorts^10^. Moreover, individually these studies are not representative of biological and clinical heterogeneity observed in the real-world patient population, which substantially limits their generalizability. The vast quantity of expression profiling data in the public repositories Gene Expression Omnibus (GEO) and ArrayExpress represents novel opportunities to address these challenges by facilitating comprehensive integration of human muscle disease cohorts for meta-analysis.

We applied a multi-cohort analysis framework^11,12^ that leverages the biological, clinical, and technical heterogeneity across independent data sets to identify a reproducible disease gene signature^13,14^. This approach has discovered robust signatures in organ rejection^13^, neurodegenerative diseases^14^, sepsis^15^, tuberculosis^16^, viral infections^17^, vaccination^18^, and systemic sclerosis^19^, many of which have been successfully validated in prospective independent cohorts^20–22^. We hypothesized that convergent transcriptional abnormalities occur across muscle diseases regardless of the specific muscle pathophysiology and that the relative expression of these genes is associated with the degree of muscle dysfunction. To the best of our knowledge, this is the largest systematic multi-cohort analysis investigating transcriptional changes across multiple human muscle diseases.

We identified a conserved gene signature across five muscle disease categories including muscular dystrophies, inflammatory myopathies, critical illness myopathy, and chronic systemic diseases associated with muscle dysfunction such as chronic obstructive pulmonary disorder (COPD). Importantly, we validated the discriminatory power of this signature in other diseases with muscle phenotypes that were not part of the discovery meta-analysis, cerebral palsy (CP) and amyotrophic lateral sclerosis (ALS). We found that the common muscle disease gene signature is significantly associated with clinical and histological disease severity in independent validation cohorts. Finally, we identified patterns of gene dysregulation unique to each muscle disease category relative to the others.

Portions of this manuscript, including the methods section, have been presented previously reported in a PhD thesis by the first author^1^. The present study has an expanded number of patient cohorts that were not included in the PhD thesis.

## Results

### Meta-analysis identifies a common gene signature of muscle diseases

A total of 45 independent patient cohorts that profiled human muscle diseases and normal muscle controls (862 cases, 512 controls), comprising 1374 samples met criteria for inclusion (Supplementary Fig. 1, Table 1). Collectively, the cohorts represent a broad range of patient ages and peripheral muscles from both upper and lower extremities. Available phenotypic data for patient samples included in public repositories is shown in Supplementary Table 1 and summary descriptions of each study are found in Supplementary Document 1.

**Table 1:** Summary of public gene expression-based discovery and validation data sets used in the meta-analysis.

Disease category	Accession#	Reference	Cases	n cases	n control	Total samples	Platform
**Discovery**							
ICUAW	GSE13205	Fredriksson^49^	Sepsis MODS	13	8	21	GPL570
ICUAW	GSE53702	Langhans^50^	ICUAW	7	6	13	GPL5188
ICUAW	GSE3307	Bakay^51^	ICUAW	5	13^c^	18^c^	GPL96
Congenital	GSE15090	Arashiro^52^	FSHD	5	5	10	GPL570
Congenital	GSE18715	Voets ^a^	POLG1	6	12	18	GPL570
Congenital	GSE36398a^b^	Rahimov^53^	FSHD	8	16	24	GPL6244
Congenital	GSE36398b	Rahimov^53^	FSHD	10	8	18	GPL6244
Congenital	GSE37084	Perfetti^54^	MMD	10	10	20	GPL5175
Congenital	GSE26852	Tasca^55^	FSHD, dysferlinopathy	12	7	19	GPL6947
Congenital	GSE47968	Nakamori^56^	FSHD, DM	23	8	31	GPL5188
Congenital	GSE42806	Screen^57^	TMD	7	5	12	GPL570
Congenital	GSE38417	Dorsey ^a^	DMD	16	6	22	GPL570
Congenital	GSE38680b	Palermo^58^	GSD II	9	10	19	GPL570
Congenital	GSE11681	Saenz^59^	LGMD2A	10	10	20	GPL96
Congenital	GSE12648	Eisenberg^60^	HIBM	10	10	20	GPL96
Congenital	GSE6011	Pescatori^61^	DMD	23	14	37	GPL96
IM	GSE48280	Surez-Calvet^62^	PM, IBM, DM	14	5	19	GPL6244
IM	GSE3307	Bakay^51^	Juvenile DM	21	13^c^	34^c^	GPL96
IM	GSE1551	Greenberg^63^	DM	13	10	23	GPL96
IM	GSE26852	Tasca^55^	PM, IM, DM	7	7	14	GPL6947
IM	EMEXP2681	Bernasconi ^a^	DM, PM	8	7	15	GPL96
Immobility	GSE45745	Barres^64^	Morbid obesity	5	6	11	GPL13667
Immobility	GSE21496	Reich^65^	Unloading	7	7	14	GPL570
Immobility	GSE5110	Urso^66^	Immobility	5	5	10	GPL570
Immobility	GSE24215	Alibegovic^67^	Immobility	12	12	24	GPL6480
Immobility	GSE104999	Rullman^68^	Immobility	12	12	24	GPL17692
Immobility	GSE474	Park^69^	Morbid obesity	16	8	24	GPL96
Chronic	GSE27536	Turan^70^	COPD	30	24	54	GPL570
Chronic	GSE1786	Radom-Aizik^71^	COPD	12	12	24	GPL96
Chronic	EMTAB3671	Kreiner^72^	PMR	12	12	24	GPL570
Total				348	288	636	
**Validation**							
ICUAW	GSE78929	Walsh^8^	ICUAW	24	8	32	GPL10558
Congenital	GSE13608	Bachinksi^73^	DMD, MMD	59	9	68	GPL570
Congenital	GSE38680a	Palermo^58^	GSD II	32	7	39	GPL570
Congenital	GSE109178	Dadgar^9^	MD	42	6	48	GPL570
Congenital	GSE3307	Bakay^51^	MD	66	13^c^	79^c^	GPL570
Congenital	GSE10760	Osborne^74^	FSHD	38	60	98	GPL96
IM	GSE3112	Greenberg^75^	PM, IBM	29	11	40	GPL96
IM	GSE39454	Zhu^76^	PM, IBM, NM	31	5	36	GPL570
Immobility	GSE14901	Abadi^30^	Limb disuse (casting)	48	24	72	GPL570
Immobility	GSE45462	Chen^77^	Limb disuse (casting)	16	16	32	GPL570
Chronic	GSE34111	Gallagher^78^	Cancer	12	6	30	GPL570
Chronic	GSE100281	Willis-Owen^79^	COPD	80	15	85	GPL11532
Total				477	180	657	
**Secondary validation**							
MND	EMEXP3260	Pradat^7^	ALS	9	10	19	GPL96
MND	GSE3307	Bakay^51^	ALS	9	13^c^	22^c^	GPL96
CP	GSE31243	Smith^80^	CP	20	20	40	GPL570
Total				38	43	81	

*IM* inflammatory myositides, *MMD* myotonic muscular dystrophy, *MD* muscular dystrophies, *DMD* Duchene’s muscular dystrophy, *FSHD* fascioscapulohumoral muscular dystrophy, *LGMD2A* limb-girdle muscular dystrophy type 2A, *HIBM* heritable IBM, *POLG1, MD, TMD* tibial muscular dystrophy, *IBM* inclusion body myositis, *DM* diabetes mellitus, *GSD II* glycogen storage disease type II, also called Pompe disease, *POLG1* mitochondrial DNA polymerase γ, *ICUAW* intensive care unit acquired weakness, *MODS* multi-organ dysfunction syndrome.

^a^Not published yet.

^b^Deltoid muscle samples removed as FSHD typically affects biceps.

^c^Same healthy controls used in subcohorts of GSE3307.

For the discovery cohort we ensured that there were at least three cohorts for each disease category that met our inclusion criteria. As there were only two cohorts for the MND category, this was not included in the discovery cohort as a disease category; instead, the two MND cohorts were included in the secondary validation cohort.

We chose smaller patient cohorts (< 30 samples) for the discovery meta-analysis and reserved larger patient cohorts and/or cohorts with clinical measures of muscle mass or strength or histologic assessments for validation analysis. For the discovery meta-analysis, 30 patient cohorts (348 cases, 288 controls) containing at least three cohorts from each of the five muscle disease categories were analyzed.

To identify the most robust differentially expressed (DE) genes across muscle diseases measured on multiple different microarray platforms we performed gene expression meta-analysis^10,11^ using a “leave-one-disease-out” strategy to correct for heterogeneity in genes DE between muscle disease categories and to avoid one muscle disease influencing the overall analysis, as described before^13,14^. We identified 209 genes that remained significantly DE in all 5 iterations of the “leave-one-disease-out” analysis (Supplementary Table 2).

**Figure 1:**
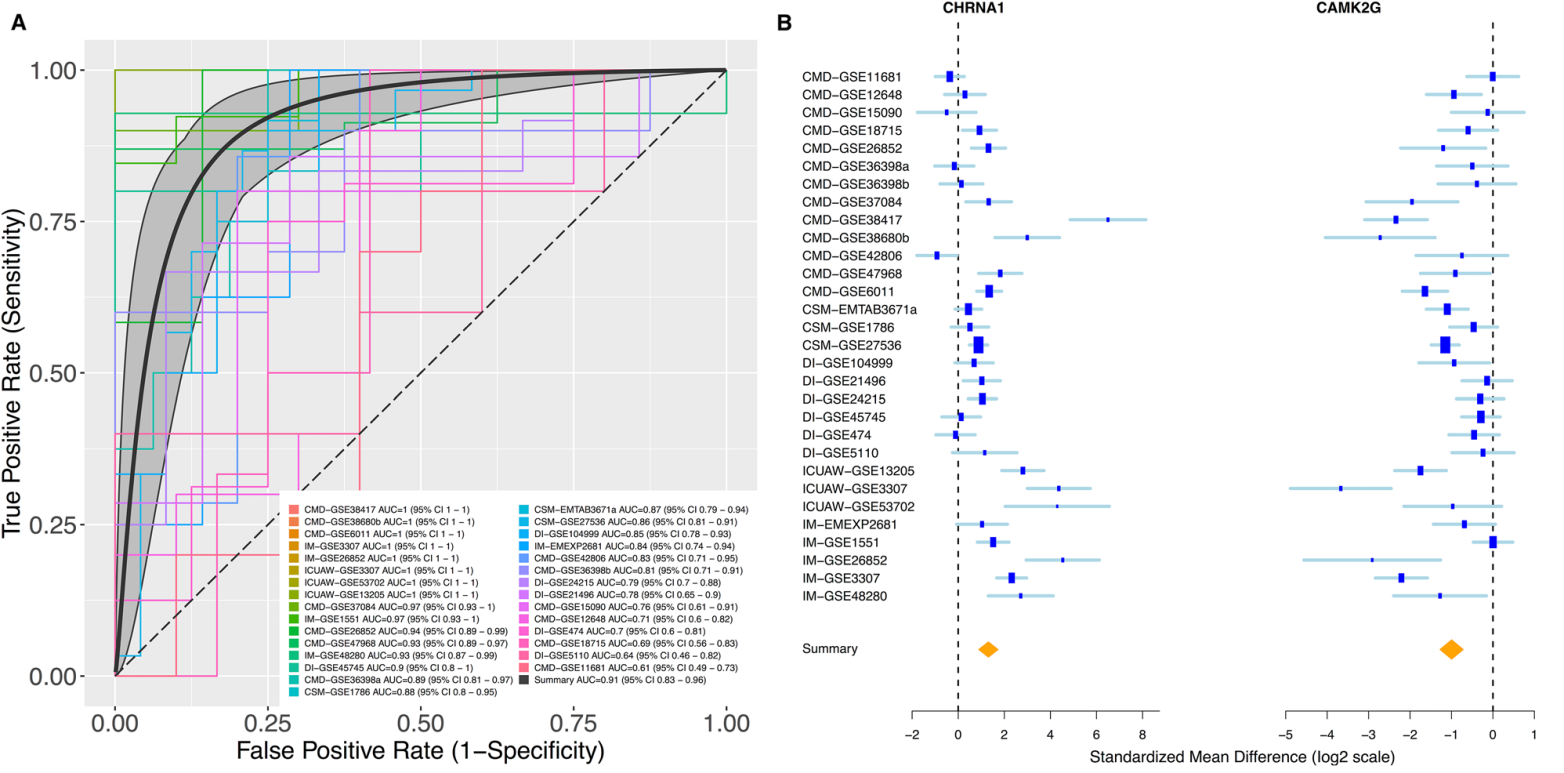
Discovery of the 52-gene signature expressed across human muscle diseases. (**A**) Meta-analysis and leave-one-disease-out analysis reveal common differentially expressed genes across muscle diseases. (**B**) Representative forest plots of most up-regulated (CHRNA1, left) and most-downregulated (CAMK2II, right) across muscle disease. The x axis represents standardized mean difference (Hedges’ g in z-scaled log 2 values) between muscle disease and controls. Summary effect sizes for each gene across all cohorts are represented as a yellow diamond.

We then applied an iterative greedy forward search^15^ to the 209 genes and identified a set of 52 genes (36 up-regulated, 16 down-regulated) that was optimized for discriminatory power termed the Common Muscle disease Module (CMDM). As expected, the 52-gene CMDM score distinguished muscle disease from healthy controls with summary area under the curve (AUC) = 0.91 (95% confidence interval [CI] 0.83- 0.96) in the discovery cohorts (Fig. 1A,B).

**Figure 2:**
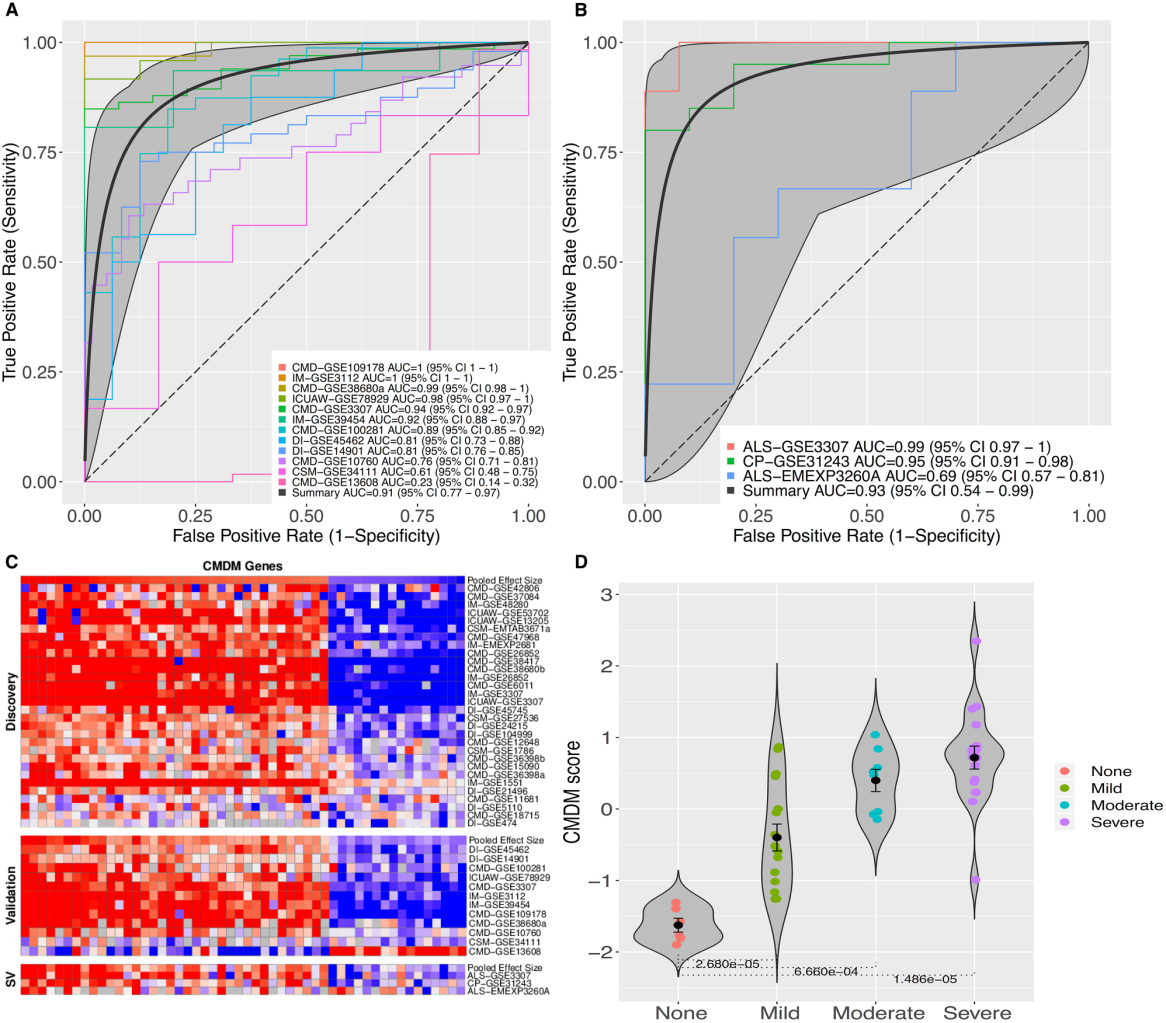
Validation of the 52-gene set of genes expressed across human muscle diseases. (**A**) ROC curves comparing 657 patients in the validation cohort. (**B**) ROC curves comparing 81 patients in the secondary validation cohort. (**C**) Heat map shows consistent differential expression in the majority of discovery, validation, and secondary validation cohort data sets. Columns represent CMDM genes ranked from the highest to the lowest standardized mean difference (Hedges’ g in z-scaled log 2 values) from left to right. Rows denote data sets used in each stage of meta-analysis, arranged by unsupervised hierarchical clustering using Ward’s minimum variance method. (**D**) Violin plots of CMDM muscle disease severity scores for a cohort of congenital muscle diseases, classified by degree of fibrosis (none, mild, moderate, severe), GSE109178. Error bars show middle quartiles. P values calculated with Wilcoxon rank-sum test. Jonckheere's trend test shows significant association (two-tailed p ≤ 0.05) p = 4.45 × 10^–9^. Refer to Table 1 for data set information. *ICUAW* intensive care unit acquired weakness, *IM* inflammatory myopathies, *DI* disuse and immobility, *CMD* congenital muscle disorders, *CSM* chronic systemic diseases affecting muscle. Genes unavailable for a dataset are shown in grey.

Next, we tested the CMDM 52- gene signature in the validation dataset of 12 cohorts (N = 657 total samples; Table 1). The validation set included at least one cohort belonging to each of the five muscle disease categories. (Table 2; Supplementary Table 3). The CMDM accurately identified muscle disease samples in most cohorts (summary AUC = 0.91 [95% CI 0.77–0.97]) (Fig. 2A).

**Table 2:** Common muscle disease module (CMDM) genes.

Gene symbol	Summary effect size in discovery set	Summary effect size in validation set	Summary effect size in secondary validation set
CHRNA1	1.31	1.125	1.796
LGMN	1.27	1.14	1.114
MYH8	1.161	1.241	2.009
C1R	1.094	0.924	0.743
AKR1A1	1.041	0.879	2.603
CDKN1A	1.04	1.009	1.855
CILP	0.993	1.189	1.01
TNFRSF21	0.992	0.942	0.464
OSBPL8	0.981	0.827	1.309
KLHL2	0.961	0.894	2.101
TMEM208	0.946	0.47	0.83
TMEM87A	0.94	0.49	0.671
IFITM2	0.932	0.599	0.195
C3	0.892	1.083	0.801
DUSP22	0.88	0.635	0.07
DDOST	0.855	0.308	0.321
LETMD1	0.847	0.844	0.563
CETN2	0.835	0.712	0.792
GPX3	0.833	0.549	− 0.252
ITPA	0.821	0.468	0.865
CLTC	0.808	0.633	0.894
SCPEP1	0.796	0.767	0.099
HEXA	0.789	0.54	− 0.145
SAE1	0.787	0.549	1.234
CHI3L1	0.765	0.442	− 0.44
USP3	0.76	0.386	0.619
HSP90B1	0.754	0.373	0.169
CKAP4	0.752	0.46	0.638
FST	0.747	0.923	1.546
NIP7	0.742	0.243	− 0.076
PANX1	0.737	0.666	0.389
HEY1	0.737	0.37	0.828
TBC1D16	0.726	0.571	0.84
TRMT112	0.711	0.475	0.698
TPP1	0.698	0.586	− 0.092
CERS2	0.673	0.158	0.793
ATP2A1	− 0.696	− 0.192	− 1.703
CS	− 0.71	− 0.673	0.185
ALDOA	− 0.719	− 0.428	− 1.847
SH2B2	− 0.721	− 0.724	− 0.573
PTP4A1	− 0.75	− 0.568	− 0.259
ACADSB	− 0.751	− 0.356	− 0.814
FXYD1	− 0.754	− 0.534	0.013
ATP5F1D	− 0.762	− 0.603	− 0.03
CAPN3	− 0.774	− 0.525	0.399
DUSP26	− 0.792	− 0.661	− 0.388
SLC25A12	− 0.834	− 0.479	− 0.32
TAPT1	− 0.865	− 0.37	− 0.506
GOT2	− 0.88	− 0.612	− 0.3
PYGM	− 0.956	− 0.432	− 1.556
LRRC20	− 0.968	− 0.635	− 0.208
CAMK2G	− 0.996	− 0.652	− 0.445

Genes are listed from the largest absolute meta-effect size to the smallest (from summary effect size in discovery set).

An additional three cohorts (two cohorts from amyotrophic lateral sclerosis [ALS] and one cohort from cerebral palsy [CP]) could not be classified into any of the five muscle disease categories present in the discovery and validation sets. Therefore we tested these three cohorts (N = 81 total samples; Table 1) as a secondary validation set to assess the generalizability of the CMDM. The CMDM accurately identified muscle disease samples in two of the three cohorts (ALS-GSE3307 and GSE31243); summary AUC of all three cohorts = 0.92 [95% CI 0.54–0.99]) (Fig. 2B).

Visual inspection of the heatmap of the 52 CMDM genes in Fig. 2C shows the pattern of expression is generally highly consistent between the discovery and validation set, as well as the secondary validation set, further supporting the generalizability of the CMDM to muscle diseases. One notable exception was the cohort GSE13608 in the validation analysis with AUC 0.23 (95% CI 0.14–0.32), which was reflected in the heatmap showing gene expression opposite to the majority of genes across the meta-analysis.

### CMDM score significantly associates with clinical and histological measures of disease severity

When selecting differentially expressed genes using the multi-cohort analysis, we did not consider disease severity. Every sample was classified as either “control” or “case.” As muscle disease severity exists along a continuum, we hypothesized that the summary expression of the CMDM would correlate with the severity of muscle disease and clinical measures of muscle function. We calculated and then correlated CMDM scores for each cohort to measures of disease severity and extent, muscle mass, strength, and function, when this detail was provided.

**Figure 3:**
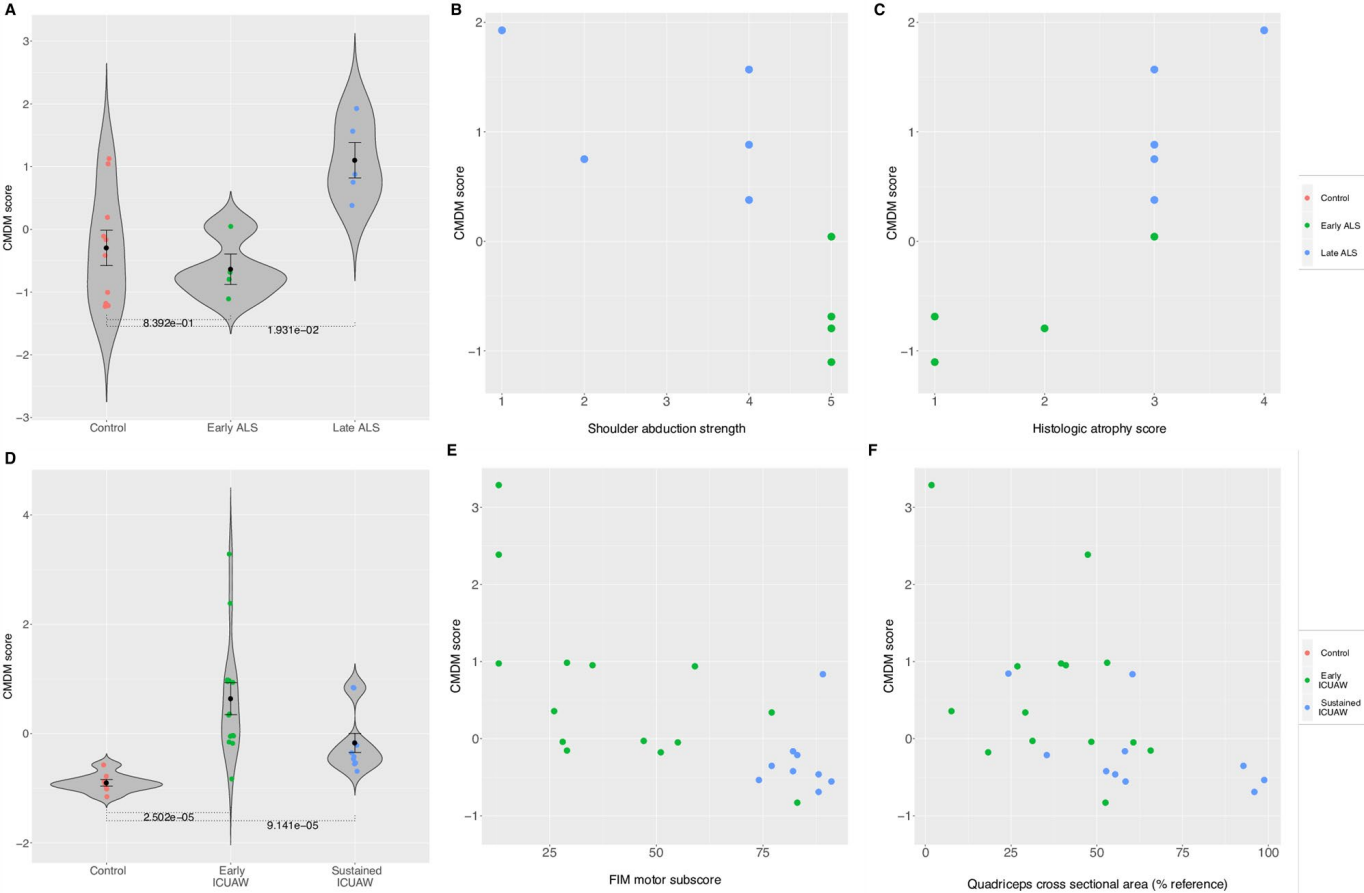
CMDM score significantly associates with clinical and histological severity in ALS and ICUAW. Plots of (**A**) CMDM scores in violin plots for controls, early ALS, and late ALS (EMEXP3260) Error bars show middle quartiles. P-values calculated with Wilcoxon rank-sum test. (**B**) Grading score of muscle atrophy in ALS (based on histology) versus CMDM score. (**C**) Shoulder abduction (muscle strength) versus CMDM score. (**D**) CMDM scores in violin plots for controls, early ICUAW (Day 7 post-ICU), and sustained ICUAW (month 6 post-ICU; GSE78929). (**E**) CMDM scores versus functional independence measure [FIM] motor subscore. (**F**) CMDM score versus quadriceps muscle mass. Each dot corresponds to individual samples. *ICUAW* ICU acquired weakness, *ALS* acute amyotrophic lateral sclerosis.

Five of the cohorts reported disease severity scores and/or clinical measures of muscle mass, strength and function. The cohort GSE109178 assessed the degree of muscle fibrosis histologically in patients with dystrophic subtypes of CMD, and categorized cases into subgroups of normal, mild, moderate, or severe fibrosis (Fig. 2D, Supplementary Table 1). Cohorts GSE78929 (ICUAW), GSE34111 (CSM) and EMEXP3260 (ALS), reported strength, muscle mass and/or physical functional capacity and the ALS cohort was additionally classified as “early” vs “late” disease severity. We found CMDM summary expression scores were significantly correlated to the histologic measures of disease severity and clinical measures of muscle mass, strength and function (Table 3 and Fig. 3A–F).

**Table 3:** Associations of common muscle disease module (CMDM) scores with clinical and histological measures of disease severity.

Cohort	Disease category	Clinic measure correlated to CMDM score	Correlation *r*	p-value
GSE109178	Congenital	Mild fibrosis vs no fibrosis	–	2.68 × 10^–5^
GSE109178	Congenital	Moderate fibrosis vs no fibrosis	–	6.66 × 10^–4^
GSE109178	Congenital	Severe fibrosis vs no fibrosis	–	1.49 × 10^–5^
EMEXP3260	MND	Muscle atrophy	0.88	1.62 × 10^–3^
EMEXP3260	MND	Muscle strength	−0.77	0.014
EMEXP3260	MND	Early ALS vs controls	–	0.84
EMEXP3260	MND	Late ALS vs controls	–	0.019
GSE78929	ICUAW	Functional independence measure (FIM) motor subscore	−0.59	3.10 × 10^–3^
GSE78929	ICUAW	Quadriceps muscle mass	−0.50	0.011
GSE78929	ICUAW	Early ICUAW (day 7 post-ICU discharge) vs controls	–	2.50 × 10^–5^
GSE78929	ICUAW	Sustained ICUAW (month 6 post-ICU discharge ) vs controls	–	9.14 × 10^–5^
GSE34111	Chronic	Quadriceps muscle strength	−0.09	0.73

### Meta-analysis highlights common mechanisms of muscle diseases

We next sought to identify conserved pathways dysregulated across muscle diseases using meta-analysis. Gene Set Enrichment Analysis (GSEA) evaluated the enrichment of Gene Ontology (GO) terms in the complete ranked list of genes based on expression relative to controls from both discovery and validation cohorts combined. A total of 74 GO Biological Process (BP) terms were significantly enriched (FDR q-value < 0.05) after removing redundant GO terms (Supplementary Table 4). Twelve GO terms were down-regulated and 62 up-regulated. Networks of overlapping significantly enriched up- and down-regulated GO terms were visualized to aid in the interpretation of the GO enrichment results (Supplementary Fig. 3).

The most down-regulated and up-regulated gene sets based on enrichment score (ES) were *regulation of skeletal muscle adaptation* (ES = -0.80, q-value = 7.9 × 10^–4^) and *macrophage migration* (ES = 0.73, q-value = 7.3 × 10^–4^), respectively. Nine of the 12 down-regulated gene sets were related to mitochondrial metabolism, including *2-oxoglutarate metabolism* and *synthesis of (ubi)quinone*, a redox-active lipid that participates in several processes including mitochondrial electron transport. Four upregulated gene sets were related to collagen metabolism and extracellular structure organization. Forty-four upregulated gene sets were related to immune system processes including *neutrophil activation*, *innate immune response* and *antigen processing*.

### Disease-specific patterns of gene expression changes

**Figure 4:**
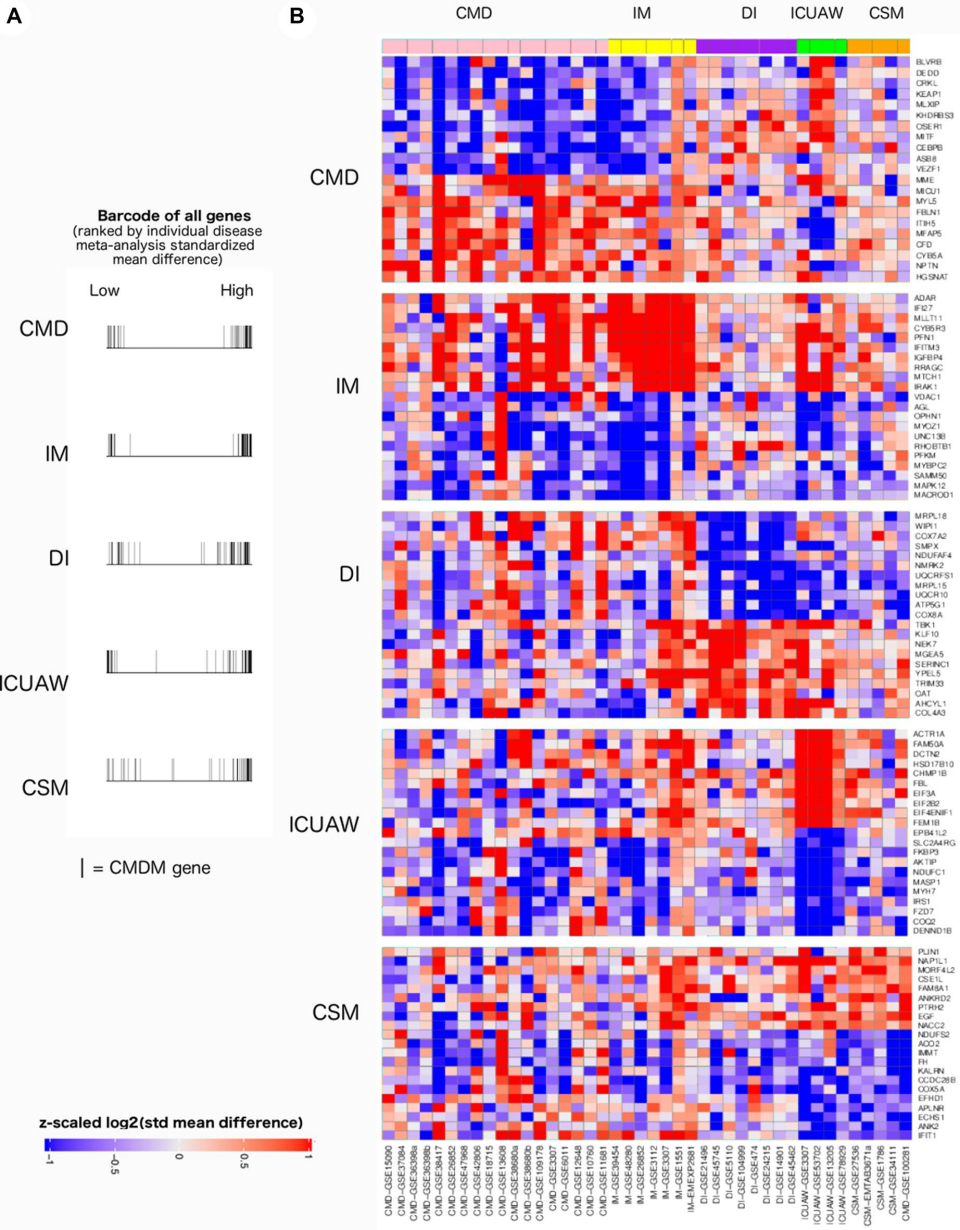
Disease-specific meta-analysis. (**A**) Distribution of the 52 CMD genes among individual disease meta-analysis gene lists. Each line presents the presence of a CMDM gene among the 24,572 gene probes generated from disease-specific meta-analysis ranked from the most positive standardized mean difference (left) to the most negative standardized mean difference (right). (**B**) Disease-specific meta-analysis after removing genes differentially expressed across the other four disease categories, identifies genes more strongly expressed in a single disease. Top 10 up-regulated and top 10 down-regulated genes shown (if 20 or more genes present). *ICUAW* intensive care unit acquired weakness, *IM* inflammatory myopathies, *DI* disuse and immobility, *CMD* congenital muscle disorders, *CSM* chronic systemic diseases affecting muscle.

We hypothesized that functional analysis of a disease category-specific gene signature, after removing genes shared with other disease categories, would provide insights into the unique pathomolecular mechanisms underlying each individual muscle disease. Thus, for each disease category we used the meta-analysis approach to generate a rank ordered list of genes based on expression relative to controls for the combined discovery and validation cohorts. We next visually examined the location of each of the CMDM genes, within the ordered list of genes, for each disease category (Fig. 4A). The CMDM genes were more densely distributed amongst the most up- and down-regulated genes for each muscle-specific category gene list, validating that the CMDM genes are similarly dysregulated in each muscle disease.

We then utilized the “leave-one-disease-out” meta-analysis approach to iteratively generate ranked lists containing four of the five disease categories. Significantly DE genes identified in the four-disease meta-analysis gene list were then removed from the single disease category meta-analysis gene list. We removed 359, 99, 200, 484, and 365 genes from the gene lists for CMD, IM, ICUAW, DI, and CSM, respectively.

The disease-specific gene lists represent genes that are expressed more strongly in a specific muscle disease category (Fig. 4B). The gene lists were then assessed for GO term enrichment to identify disease-specific pathways (Supplementary Fig. 4A–E. Supplementary Table 5A–E). Despite removing genes significant to other disease categories, persistent down-regulation of (i) gene sets related to mitochondrial electron transport were found in IM, DI, ICUAW, and CSM cohorts and (ii) gene sets related to mitochondrial translation were found in CMD, DI, and CSM cohorts. Significant down-regulation of genes sets related to muscle contraction were identified in CMD, IM, and ICUAW. Upregulation of mRNA splicing via spliceosome was found in ICUAW and DI. Upregulation of extracellular matrix organization genes were observed in CMD and IM. Significant up regulation of NF-kB signaling genes was found in IM and ICUAW categories. Only ICUAW had down-regulation of genes related to cell fate specification, including SOX17, a transcription factor induced during satellite cell specification^23^.

### Subcellular localization analysis of CMDM genes

We explored whether the CMDM gene signature is overrepresented in certain subcellular compartments. The majority of CMDM genes (98.1%) mapped to at least one subcellular localization. The vesicular exosome contained the greatest proportion of CMDM genes (25%) and was significantly overrepresented in CMDM signature (q-value = 0.039) (Supplementary Table 6A and 6B, Supplementary Fig. 5). One gene (*FST)* was extracellular.

## Discussion

We analyzed the reported transcriptomes of 1374 individual muscle samples collected from 45 independent patient/control cohorts classified into five categories of skeletal muscle disease and derived using multiple microarray platforms, to identify and validate a robust and reproducible gene signature of muscle disease. This analysis leveraged both biological and technical heterogeneity across multiple independent cohorts in the discovery cohort to avoid overfitting and validated the CMDM signature using cohorts containing larger sample sizes to reduce technical heterogeneity^11^. To assess the generalizability of the signature we examined three more cohorts that could not be classified within the five specified muscle disease categories and found the signature to be reproducible in these cohorts as well.

Although there are heterogeneous muscle types and diverse genetic and acquired causes of different muscle diseases, the 52-gene CMDM reflected convergent transcriptional pathways across peripheral skeletal muscles affected by disease. This implies that prior characterization of any of the genes in the CMDM may be relevant across muscle diseases. Several of the genes in the CMDM have been associated with muscle disease previously, whereas many remain unknown or poorly characterized in skeletal muscle.

Increased expression of cholinergic receptor nicotinic, alpha 1 (*CHRNA1*), the most robustly DE CMDM gene, has been recognized as a marker of severity of muscle denervation^24,25^*.* Up-regulation of *CHRNA1* has been reported to be associated with dynamic epigenetic modifications of the gene in a rat model of disuse-induced atrophy^26^. The most down-regulated CMDM gene CAMK2G, calcium/calmodulin-dependent protein kinase type II (CaMKII) subunit gamma, is involved in sarcoplasmic reticulum Ca2+ transport in skeletal muscle and has been shown to remain active after exercise^27^. While agonists of CaMKII have been proposed as potential pharmacologic therapies of in various muscle disease^28^ , it has remained unclear which of the CaMKII subunits is most important in the regulation of skeletal muscle adaptation, response to injury and activity, and oxidative capacity as these subunits are currently not well characterized. Given that CAMK2G is down-regulated across most muscle diseases in this study we propose that it may be a suitable target for future studies of potential therapeutics.

Although able to robustly identify a broad range of muscle diseases, the CMDM signature more importantly strongly correlates with clinical and histological measures of disease severity, providing persuasive evidence that the signature could have future applications as a biomarker for phenotyping muscle disease. The CMDM signature could specifically provide diagnostic information and quantify the molecular response to therapy for muscle disease. Measuring changes in CMDM scores after treatment may improve the identification of therapy responders, and using it at enrollment in therapeutic trials may aid the stratification of patients within trial arms. Furthermore this signature could also serve to phenotype patients with COPD, ICUAW and other chronic respiratory diseases based on the extent of muscle dysfunction.

We applied gene set enrichment analysis to identify functional pathways that are similarly altered across muscle diseases. As expected, genes involved in skeletal muscle skeletal adaptation and mitochondrial function were down-regulated. Coordinate down-regulation of mitochondrial genes has been described in a number of muscle diseases^29–32^. The predominant up-regulated functional terms were related to immune activation. Muscle damage secondary to disease induces immune activation culminating in inflammation and deposition of extracellular matrix (ECM)^33,34^. Skeletal muscle diseases are characterized by up-regulation of ECM genes including collagen, with progressive development of fibrosis leading to dysfunctional muscle tissue^35,36^. Collectively, these findings are consistent with literature in chronic skeletal muscle diseases proposing the convergence of final common pathways including chronic inflammation, fibrosis, oxidative stress, and mitochondrial dysfunction^36^.

We next removed genes similarly dysregulated across muscle disease to identify pathways altered within specific muscle diseases. This strategy identified pathways unique to ICUAW as well as those shared with other muscle diseases. Significant up-regulation of NF-*k*B signaling genes was found in IM and ICUAW. NF-*k*B has been previously shown to play a role in IM^37,38^, has been studied in animal models of cancer cachexia and ICUAW^39–41^ and has been shown to be an inhibitor of skeletal myogenesis and muscle regeneration^42^. Remarkably, only ICUAW had down-regulation of genes related to cell fate specification. Decreased numbers of satellite cells (precursors to skeletal muscle cells) in ICUAW sustained long term, compared to healthy controls have been detected histologically^43^, supporting the finding of a down-regulated stem-cell gene set.

An unexpected finding of our meta-analysis was that the CMDM signature is enriched for genes targeted to the exosomal vesicle. Vesicular exosomes, cell derived vesicles containing signaling factors (including genes and microRNAs) for intercellular communication, have been found to have roles in muscle regeneration and congenital muscle diseases^44^. Monitoring exosomal miRNAs has been proposed as a non-invasive method for tracking muscle disease progression^44,45^. Future studies will assess whether plasma protein concentrations of the exosomal CMDM genes correlate with muscle severity to the same extent as their transcripts.

Our meta-analysis has limitations despite its comprehensiveness. Although most included studies attempted to select patients without co-morbidities that span more than one muscle disease category, there are potentially multiple pathologies in some of the muscle samples. Given the number of cohorts and size of the overall study, such confounding is likely to be minimal. The broad inclusion criteria applied in this study has identified a robust disease signature that reflects the heterogeneity observed in the real-world patient population. The considerable variance in gene expression profiles between the different muscle tissue sites^46^ included in this analysis is expected to have reduced the number of significant genes, while increasing the generalizability of the significant genes detected. We primarily focused on identifying a gene signature that is conserved between several muscle disease categories and across samples. Although this is beneficial for capturing features that are consistent across multiple diseases, it is ill-suited for identifying subgroups of disease.

Based on the use of microarray data from multiple platforms, we cannot test for alterations in splicing regulation, which has been associated with several congenital muscle diseases including the most common adult onset muscular dystrophies^47^. Analysis of RNA-seq transcriptome data will be necessary to determine whether altered splice variants lead to muscle pathology in other disease categories. Identification of conserved epigenetic signatures of muscle disease will provide important insights into the underlying mechanisms resulting in gene transcriptomic dysregulation identified here, once future epigenome-wide association studies of various muscle diseases are available.

The cohort GSE34111 had a global expression pattern that differed markedly from the other muscle diseases and disease categories. As this cohort was the only one in the analysis that included cancer cachexia, it remains unclear whether the difference in global expression pattern reflects significant differences in the pathomechanism of cancer cachexia or technical or experimental differences in the study. Future analysis comparing peripheral muscle from patients with cancer cachexia and controls are required. Within the validation set, the chronic systemic disease and ICUAW categories each consisted of one cohort, reducing the power to detect significant effect size differences from controls within these disease categories. For this reason, disease specific pathway analysis was performed by combing both discovery and validation cohorts.

CMDM genes may be conceptually divided into those having direct etiological contribution to muscle disease and those that represent a secondary phenomenon in the development of muscle disease, include stress-related changes or cell survival mechanisms^48^. Further experimentation will be required to identify the CMDM genes directly contributing to disease as these genes are expected to be good candidates for novel disease modifying therapies^14^. CMDM genes without functional annotation can be prioritized for future experimental evaluation based on the strength of the molecular data (e.g. effect size or correlation with clinical phenotype). Direct experimentation will be necessary to determine the role of the dysregulated genes and pathways in muscle disease as either causal drivers or responses to muscle disease.

Our results identify a conserved muscle disease transcriptional signature associated with clinical and histologic disease severity, and identify numerous novel genes associated with muscle disease severity. Muscle disease specific analysis identifies pathways uniquely altered in ICUAW. Thus our findings serve as a valuable resource for interpreting disease mechanisms, connecting findings across muscle diseases, and driving novel hypotheses.

## Methods

The analysis workflow is shown in Supplementary Fig. 1.

### Data collection and pre-processing

Two public gene expression microarray repositories (ArrayExpress, NIH GEO) were searched for human muscle disease datasets (search date: Aug 29, 2019). We found 45 independent datasets with 1374 muscle biopsies that met our inclusion criteria (Supplementary Methods).

We divided the sample cohorts into 6 disease categories for analysis: (1) inflammatory myopathies (IM), (2) ICU acquired weakness (ICUAW), (3) congenital muscle diseases (CMD), (4) chronic systemic disease affecting muscle (CSM), (5) disuse and immobility (DI), (6) motor neuron disease (MND). Next, we divided the patient cohorts into a *discovery cohort* for the initial meta-analysis and a validation cohort for the independent validation analysis. For the discovery cohort we ensured that there were at least three cohorts for each disease category that met our inclusion criteria. As there were only two cohorts for the MND category, this was not included in the discovery cohort as a disease category; instead, the two MND cohorts were included in the secondary validation cohort.

Normalization and probe expression summarization are described in Supplementary Methods. The number of studies measured for each gene are listed in Supplementary Tables 2 and 3.

### Meta-analysis

Multicohort meta-analysis of gene expression was performed (using R package *MetaIntegrator*)^12^ as described in the Supplementary Methods. The utility of the leave-one-disease-out approach in identifying a robust gene expression signature during acute rejection across different transplanted solid organs^13^ and across neurodegenerative diseases^14^ has been shown before.

### Derivation of common muscle disease module (CMDM) score

We applied a greedy forward search as described in the Supplementary Methods section to identify a gene signature maximized for diagnostic power, termed the Common Muscle Disease Module (CMDM).

### Validation of CMDM score and correlation of the CMDM genes with clinical and histological severity

Tukey’s Biweight correlation was used to assess the association of the CMDM score with the histologic and clinical measures. Between- and within-group CMDM score comparisons were done with the Wilcoxon rank sum test.

### Muscle disease category specific meta-analysis

To identify patterns on gene expression changes that are unique to each muscle disease category we performed meta-analysis using the combined data of the discovery and validation cohorts. We first analyzed each disease category separately, as well as the other four diseases together.. Genes that were significantly differentially expressed in the four-disease category meta-analysis were then removed from the individual disease category meta-analysis, thereby removing the DE genes common to all muscle disorders, from the disease-specific gene list.

Gene ontology functional analysis identified functional themes within differentially expressed genes across muscle disease categories as described in the Supplementary Methods*.*

Subcellular localization analysis was performed for each gene within the CMDM as described in the Supplementary Methods.

All analyses were completed in R language for statistical computing (version 3.4.1). Significance levels were set at two-tailed p < 0.05, unless specified otherwise.

## Supplementary Information

Supplementary Figure 1.

Supplementary Information 1.

Supplementary Legends.

Supplementary Figure 2.

Supplementary Figure 3.

Supplementary Figure 4A.

Supplementary Figure 4B.

Supplementary Figure 4C.

Supplementary Figure 4D.

Supplementary Figure 4E.

Supplementary Figure 5.

Supplementary Information 2.

Supplementary Table 1.

Supplementary Table 2.

Supplementary Table 3.

Supplementary Table 4.

Supplementary Table 5.

Supplementary Table 6A.

Supplementary Table 6B.

## Data Availability

The datasets supporting the results of this article are available in GEO and ArrayExpress online repositories at http://www.ebi.ac.uk/ arrayexpress/ and http://www.ncbi.nlm.nih.gov/geo/. Data set accession numbers can be found in Table 1.

## References

[1] Walsh, C.J. *Transcriptional Profiling and Regulation in Survivors of Critical Illness with Muscle Weakness and Meta-analysis across Human Muscle Diseases*. TSpace http://hdl.handle.net/1807/97727. (University of Toronto, 2019).

[2] Barreiro E, Sznajder JI, Nader GA, Budinger GR (2015). Muscle dysfunction in patients with lung diseases: A growing epidemic. Am. J. Respir. Crit. Care Med..

[3] Abdulai RM, Jensen TJ, Patel NR, Polkey MI, Jansson P, Celli BR, Rennard SI (2018). Deterioration of limb muscle function during acute exacerbation of chronic obstructive pulmonary disease. Am. J. Respir. Crit. Care Med..

[4] Riou M, Pizzimenti M, Enache I, Charloux A, Canuet M, Andres E, Talha S, Meyer A, Geny B (2020). Skeletal and respiratory muscle dysfunctions in pulmonary arterial hypertension. J. Clin. Med..

[5] Puthucheary ZA, McNelly AS, Rawal J, Connolly B, Sidhu PS, Rowlerson A, Moxham J, Harridge SD, Hart N, Montgomery HE (2017). Rectus femoris cross-sectional area and muscle layer thickness: Comparative markers of muscle wasting and weakness. Am. J. Respir. Crit. Care Med..

[6] Man WD, Soliman MG, Nikoletou D, Harris ML, Rafferty GF, Mustfa N, Polkey MI, Moxham J (2003). Non-volitional assessment of skeletal muscle strength in patients with chronic obstructive pulmonary disease. Thorax.

[7] Pradat PF, Dubourg O, de Tapia M, di Scala F, Dupuis L, Lenglet T, Bruneteau G, Salachas F, Lacomblez L, Corvol JC (2012). Muscle gene expression is a marker of amyotrophic lateral sclerosis severity. Neurodegener. Dis..

[8] Walsh CJ, Batt J, Herridge MS, Mathur S, Bader GD, Hu P, Dos Santos CC (2016). Transcriptomic analysis reveals abnormal muscle repair and remodeling in survivors of critical illness with sustained weakness. Sci. Rep..

[9] Dadgar S, Wang Z, Johnston H, Kesari A, Nagaraju K, Chen YW, Hill DA, Partridge TA, Giri M, Freishtat RJ (2014). Asynchronous remodeling is a driver of failed regeneration in Duchenne muscular dystrophy. J. Cell Biol..

[10] Walsh CJ, Hu P, Batt J, Santos CC (2015). Microarray meta-analysis and cross-platform normalization: Integrative genomics for robust biomarker discovery. Microarrays (Basel).

[11] Sweeney TE, Haynes WA, Vallania F, Ioannidis JP, Khatri P (2017). Methods to increase reproducibility in differential gene expression via meta-analysis. Nucleic Acids Res..

[12] Haynes WA, Vallania F, Liu C, Bongen E, Tomczak A, Andres-Terre M, Lofgren S, Tam A, Deisseroth CA, Li MD (2017). Empowering multi-cohort gene expression analysis to increase reproducibility. Pac. Symp. Biocomput..

[13] Khatri P, Roedder S, Kimura N, De Vusser K, Morgan AA, Gong Y, Fischbein MP, Robbins RC, Naesens M, Butte AJ (2013). A common rejection module (CRM) for acute rejection across multiple organs identifies novel therapeutics for organ transplantation. J. Exp. Med..

[14] Li MD, Burns TC, Morgan AA, Khatri P (2014). Integrated multi-cohort transcriptional meta-analysis of neurodegenerative diseases. Acta Neuropathol. Commun..

[15] Sweeney TE, Shidham A, Wong HR, Khatri P (2015). A comprehensive time-course-based multicohort analysis of sepsis and sterile inflammation reveals a robust diagnostic gene set. Sci. Transl. Med..

[16] Sweeney TE, Braviak L, Tato CM, Khatri P (2016). Genome-wide expression for diagnosis of pulmonary tuberculosis: A multicohort analysis. Lancet Respir. Med..

[17] Sweeney TE, Wong HR, Khatri P (2016). Robust classification of bacterial and viral infections via integrated host gene expression diagnostics. Sci. Transl. Med..

[18] Team H-CSP, Consortium H-I: Multicohort analysis reveals baseline transcriptional predictors of influenza vaccination responses. *Sci. Immunol.* **2**(14) (2017). 10.1126/sciimmunol.aal4656 PMC5800877 28842433

[19] Lofgren S, Hinchcliff M, Carns M, Wood T, Aren K, Arroyo E, Cheung P, Kuo A, Valenzuela A, Haemel A (2016). Integrated, multicohort analysis of systemic sclerosis identifies robust transcriptional signature of disease severity. JCI Insight.

[20] Maslove DM, Shapira T, Tyryshkin K, Veldhoen RA, Marshall JC, Muscedere J (2019). Validation of diagnostic gene sets to identify critically ill patients with sepsis. J. Crit. Care.

[21] Francisco NM, Fang YM, Ding L, Feng S, Yang Y, Wu M, Jacobs M, Ryffel B, Huang X (2017). Diagnostic accuracy of a selected signature gene set that discriminates active pulmonary tuberculosis and other pulmonary diseases. J. Infect..

[22] Mayhew MB, Buturovic L, Luethy R, Midic U, Moore AR, Roque JA, Shaller BD, Asuni T, Rawling D, Remmel M (2020). A generalizable 29-mRNA neural-network classifier for acute bacterial and viral infections. Nat. Commun..

[23] Alonso-Martin S, Aurade F, Mademtzoglou D, Rochat A, Zammit PS, Relaix F (2018). SOXF factors regulate murine satellite cell self-renewal and function through inhibition of beta-catenin activity. Elife.

[24] Anderson DM, Cannavino J, Li H, Anderson KM, Nelson BR, McAnally J, Bezprozvannaya S, Liu Y, Lin W, Liu N (2016). Severe muscle wasting and denervation in mice lacking the RNA-binding protein ZFP106. Proc. Natl. Acad. Sci. U S A.

[25] von Grabowiecki Y, Abreu P, Blanchard O, Palamiuc L, Benosman S, Meriaux S, Devignot V, Gross I, Mellitzer G, Gonzalez de Aguilar JL (2016). Transcriptional activator TAp63 is upregulated in muscular atrophy during ALS and induces the pro-atrophic ubiquitin ligase Trim63. Elife.

[26] Fisher AG, Seaborne RA, Hughes TM, Gutteridge A, Stewart C, Coulson JM, Sharples AP, Jarvis JC (2017). Transcriptomic and epigenetic regulation of disuse atrophy and the return to activity in skeletal muscle. FASEB J..

[27] Rose AJ, Hargreaves M (2003). Exercise increases Ca2+-calmodulin-dependent protein kinase II activity in human skeletal muscle. J. Physiol..

[28] Chin ER (2004). The role of calcium and calcium/calmodulin-dependent kinases in skeletal muscle plasticity and mitochondrial biogenesis. Proc. Nutr. Soc..

[29] Jiroutkova K, Krajcova A, Ziak J, Fric M, Waldauf P, Dzupa V, Gojda J, Nemcova-Furstova V, Kovar J, Elkalaf M (2015). Mitochondrial function in skeletal muscle of patients with protracted critical illness and ICU-acquired weakness. Crit. Care.

[30] Abadi A, Glover EI, Isfort RJ, Raha S, Safdar A, Yasuda N, Kaczor JJ, Melov S, Hubbard A, Qu X (2009). Limb immobilization induces a coordinate down-regulation of mitochondrial and other metabolic pathways in men and women. PLoS ONE.

[31] Taivassalo T, Hussain SN (2016). Contribution of the mitochondria to locomotor muscle dysfunction in patients With COPD. Chest.

[32] Temiz P, Weihl CC, Pestronk A (2009). Inflammatory myopathies with mitochondrial pathology and protein aggregates. J. Neurol. Sci..

[33] Dumont N, Bouchard P, Frenette J (2008). Neutrophil-induced skeletal muscle damage: A calculated and controlled response following hindlimb unloading and reloading. Am. J. Physiol. Regul. Integr. Comp. Physiol..

[34] Madaro L, Bouche M (2014). From innate to adaptive immune response in muscular dystrophies and skeletal muscle regeneration: The role of lymphocytes. Biomed. Res. Int..

[35] Gillies AR, Chapman MA, Bushong EA, Deerinck TJ, Ellisman MH, Lieber RL (2017). High resolution three-dimensional reconstruction of fibrotic skeletal muscle extracellular matrix. J. Physiol..

[36] Mann CJ, Perdiguero E, Kharraz Y, Aguilar S, Pessina P, Serrano AL, Munoz-Canoves P (2011). Aberrant repair and fibrosis development in skeletal muscle. Skelet. Muscle.

[37] Monici MC, Aguennouz M, Mazzeo A, Messina C, Vita G (2003). Activation of nuclear factor-B in inflammatory myopathies and Duchenne muscular dystrophy. Neurology.

[38] Yang C-C, Askanas V, Engel WK, Alvarez RB (1998). Immunolocalization of transcription factor NF-κB in inclusion-body myositis muscle and at normal human neuromuscular junctions. Neurosci. Lett..

[39] Guttridge DC (2000). NF-kappa B-induced loss of MyoD messenger RNA: Possible role in muscle decay and cachexia. Science.

[40] Mourkioti F, Rosenthal N (2008). NF-kappaB signaling in skeletal muscle: Prospects for intervention in muscle diseases. J. Mol. Med. (Berl).

[41] Friedrich O, Reid MB, Van den Berghe G, Vanhorebeek I, Hermans G, Rich MM, Larsson L (2015). The sick and the weak: Neuropathies/myopathies in the critically ill. Physiol. Rev..

[42] Bakkar N, Guttridge DC (2010). NF-kappaB signaling: A tale of two pathways in skeletal myogenesis. Physiol. Rev..

[43] Dos Santos C, Hussain SN, Mathur S, Picard M, Herridge M, Correa J, Bain A, Guo Y, Advani A, Advani SL (2016). Mechanisms of chronic muscle wasting and dysfunction after an intensive care unit stay. A pilot study. Am. J. Respir. Crit. Care Med..

[44] Murphy C, Withrow J, Hunter M, Liu Y, Tang YL, Fulzele S, Hamrick MW (2018). Emerging role of extracellular vesicles in musculoskeletal diseases. Mol. Aspects Med..

[45] Koutsoulidou A, Kyriakides TC, Papadimas GK, Christou Y, Kararizou E, Papanicolaou EZ, Phylactou LA (2015). Elevated muscle-specific miRNAs in serum of myotonic dystrophy patients relate to muscle disease progress. PLoS ONE.

[46] Terry EE, Zhang X, Hoffmann C, Hughes LD, Lewis SA, Li J, Wallace MJ, Riley LA, Douglas CM, Gutierrez-Monreal MA (2018). Transcriptional profiling reveals extraordinary diversity among skeletal muscle tissues. Elife.

[47] Imbriano C, Molinari S (2018). Alternative splicing of transcription factors genes in muscle physiology and pathology. Genes (Basel).

[48] Cagliani R, Magri F, Toscano A, Merlini L, Fortunato F, Lamperti C, Rodolico C, Prelle A, Sironi M, Aguennouz M (2005). Mutation finding in patients with dysferlin deficiency and role of the dysferlin interacting proteins annexin A1 and A2 in muscular dystrophies. Hum. Mutat..

[49] Fredriksson K, Tjader I, Keller P, Petrovic N, Ahlman B, Scheele C, Wernerman J, Timmons JA, Rooyackers O (2008). Dysregulation of mitochondrial dynamics and the muscle transcriptome in ICU patients suffering from sepsis induced multiple organ failure. PLoS ONE.

[50] Langhans C, Weber-Carstens S, Schmidt F, Hamati J, Kny M, Zhu X, Wollersheim T, Koch S, Krebs M, Schulz H (2014). Inflammation-induced acute phase response in skeletal muscle and critical illness myopathy. PLoS ONE.

[51] Bakay M, Wang Z, Melcon G, Schiltz L, Xuan J, Zhao P, Sartorelli V, Seo J, Pegoraro E, Angelini C (2006). Nuclear envelope dystrophies show a transcriptional fingerprint suggesting disruption of Rb-MyoD pathways in muscle regeneration. Brain.

[52] Arashiro P, Eisenberg I, Kho AT, Cerqueira AM, Canovas M, Silva HC, Pavanello RC, Verjovski-Almeida S, Kunkel LM, Zatz M (2009). Transcriptional regulation differs in affected facioscapulohumeral muscular dystrophy patients compared to asymptomatic related carriers. Proc. Natl. Acad. Sci. USA.

[53] Rahimov F, King OD, Leung DG, Bibat GM, Emerson CP, Kunkel LM, Wagner KR (2012). Transcriptional profiling in facioscapulohumeral muscular dystrophy to identify candidate biomarkers. Proc. Natl. Acad. Sci. USA.

[54] Perfetti A, Greco S, Fasanaro P, Bugiardini E, Cardani R, Garcia-Manteiga JM, Riba M, Cittaro D, Stupka E, Meola G (2014). Genome wide identification of aberrant alternative splicing events in myotonic dystrophy type 2. PLoS ONE.

[55] Tasca G, Pescatori M, Monforte M, Mirabella M, Iannaccone E, Frusciante R, Cubeddu T, Laschena F, Ottaviani P, Ricci E (2012). Different molecular signatures in magnetic resonance imaging-staged facioscapulohumeral muscular dystrophy muscles. PLoS ONE.

[56] Nakamori M, Sobczak K, Puwanant A, Welle S, Eichinger K, Pandya S, Dekdebrun J, Heatwole CR, McDermott MP, Chen T (2013). Splicing biomarkers of disease severity in myotonic dystrophy. Ann. Neurol..

[57] Screen M, Raheem O, Holmlund-Hampf J, Jonson PH, Huovinen S, Hackman P, Udd B (2014). Gene expression profiling in tibial muscular dystrophy reveals unfolded protein response and altered autophagy. PLoS ONE.

[58] Palermo AT, Palmer RE, So KS, Oba-Shinjo SM, Zhang M, Richards B, Madhiwalla ST, Finn PF, Hasegawa A, Ciociola KM (2012). Transcriptional response to GAA deficiency (Pompe disease) in infantile-onset patients. Mol. Genet. Metab..

[59] Saenz A, Azpitarte M, Armananzas R, Leturcq F, Alzualde A, Inza I, Garcia-Bragado F, De la Herran G, Corcuera J, Cabello A (2008). Gene expression profiling in limb-girdle muscular dystrophy 2A. PLoS ONE.

[60] Eisenberg I, Novershtern N, Itzhaki Z, Becker-Cohen M, Sadeh M, Willems PH, Friedman N, Koopman WJ, Mitrani-Rosenbaum S (2008). Mitochondrial processes are impaired in hereditary inclusion body myopathy. Hum. Mol. Genet..

[61] Pescatori M, Broccolini A, Minetti C, Bertini E, Bruno C, D'Amico A, Bernardini C, Mirabella M, Silvestri G, Giglio V (2007). Gene expression profiling in the early phases of DMD: A constant molecular signature characterizes DMD muscle from early postnatal life throughout disease progression. FASEB J..

[62] Suarez-Calvet X, Gallardo E, Nogales-Gadea G, Querol L, Navas M, Diaz-Manera J, Rojas-Garcia R, Illa I (2014). Altered RIG-I/DDX58-mediated innate immunity in dermatomyositis. J. Pathol..

[63] Greenberg SA, Pinkus JL, Pinkus GS, Burleson T, Sanoudou D, Tawil R, Barohn RJ, Saperstein DS, Briemberg HR, Ericsson M (2005). Interferon-alpha/beta-mediated innate immune mechanisms in dermatomyositis. Ann. Neurol..

[64] Barres R, Kirchner H, Rasmussen M, Yan J, Kantor FR, Krook A, Naslund E, Zierath JR (2013). Weight loss after gastric bypass surgery in human obesity remodels promoter methylation. Cell Rep..

[65] Reich KA, Chen YW, Thompson PD, Hoffman EP, Clarkson PM (2010). Forty-eight hours of unloading and 24 h of reloading lead to changes in global gene expression patterns related to ubiquitination and oxidative stress in humans. J. Appl. Physiol..

[66] Urso ML, Scrimgeour AG, Chen YW, Thompson PD, Clarkson PM (2006). Analysis of human skeletal muscle after 48 h immobilization reveals alterations in mRNA and protein for extracellular matrix components. J. Appl. Physiol. (1985).

[67] Alibegovic AC, Sonne MP, Hojbjerre L, Bork-Jensen J, Jacobsen S, Nilsson E, Faerch K, Hiscock N, Mortensen B, Friedrichsen M (2010). Insulin resistance induced by physical inactivity is associated with multiple transcriptional changes in skeletal muscle in young men. Am. J. Physiol. Endocrinol. Metab..

[68] Rullman E, Mekjavic IB, Fischer H, Eiken O (2016). PlanHab (planetary habitat simulation): The combined and separate effects of 21 days bed rest and hypoxic confinement on human skeletal muscle miRNA expression. Physiol. Rep..

[69] Park JJ, Berggren JR, Hulver MW, Houmard JA, Hoffman EP (2006). GRB14, GPD1, and GDF8 as potential network collaborators in weight loss-induced improvements in insulin action in human skeletal muscle. Physiol. Genomics.

[70] Turan N, Kalko S, Stincone A, Clarke K, Sabah A, Howlett K, Curnow SJ, Rodriguez DA, Cascante M, O'Neill L (2011). A systems biology approach identifies molecular networks defining skeletal muscle abnormalities in chronic obstructive pulmonary disease. PLoS Comput. Biol..

[71] Radom-Aizik S, Kaminski N, Hayek S, Halkin H, Cooper DM, Ben-Dov I (2007). Effects of exercise training on quadriceps muscle gene expression in chronic obstructive pulmonary disease. J. Appl. Physiol. (1985).

[72] Kreiner FF, Borup R, Nielsen FC, Schjerling P, Galbo H (2017). Gene expression profiling in patients with polymyalgia rheumatica before and after symptom-abolishing glucocorticoid treatment. BMC Musculoskelet. Disord..

[73] Bachinski LL, Sirito M, Bohme M, Baggerly KA, Udd B, Krahe R (2010). Altered MEF2 isoforms in myotonic dystrophy and other neuromuscular disorders. Muscle Nerve.

[74] Osborne RJ, Welle S, Venance SL, Thornton CA, Tawil R (2007). Expression profile of FSHD supports a link between retinal vasculopathy and muscular dystrophy. Neurology.

[75] Greenberg SA, Bradshaw EM, Pinkus JL, Pinkus GS, Burleson T, Due B, Bregoli L, O'Connor KC, Amato AA (2005). Plasma cells in muscle in inclusion body myositis and polymyositis. Neurology.

[76] Zhu W, Streicher K, Shen N, Higgs BW, Morehouse C, Greenlees L, Amato AA, Ranade K, Richman L, Fiorentino D (2012). Genomic signatures characterize leukocyte infiltration in myositis muscles. BMC Med. Genomics.

[77] Chen YW, Gregory C, Ye F, Harafuji N, Lott D, Lai SH, Mathur S, Scarborough M, Gibbs P, Baligand C (2017). Molecular signatures of differential responses to exercise trainings during rehabilitation. Biomed. Genet. Genom..

[78] Gallagher IJ, Stephens NA, MacDonald AJ, Skipworth RJ, Husi H, Greig CA, Ross JA, Timmons JA, Fearon KC (2012). Suppression of skeletal muscle turnover in cancer cachexia: Evidence from the transcriptome in sequential human muscle biopsies. Clin. Cancer Res..

[79] Willis-Owen SAG, Thompson A, Kemp PR, Polkey MI, Cookson W, Moffatt MF, Natanek SA (2018). COPD is accompanied by co-ordinated transcriptional perturbation in the quadriceps affecting the mitochondria and extracellular matrix. Sci. Rep..

[80] Smith LR, Chambers HG, Subramaniam S, Lieber RL (2012). Transcriptional abnormalities of hamstring muscle contractures in children with cerebral palsy. PLoS ONE.

