# Supplementary figures and images for: Comprehensive multi-cohort transcriptional meta-analysis of muscle diseases identifies a signature of disease severity
Source: Sci Rep. 2022 Jul 4;12:11260. doi: 10.1038/s41598-022-15003-1 (PMC9253003; doi:10.1038/s41598-022-15003-1)

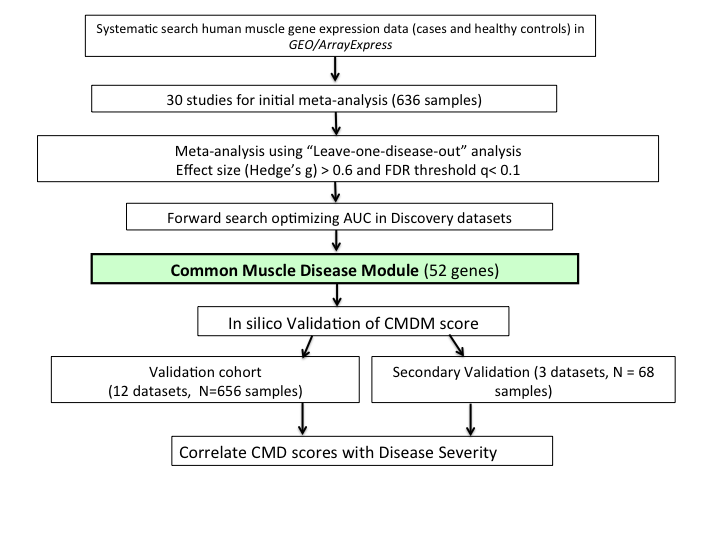

Supplement: Supplementary file 1 — Supplementary Figure 1. [file 41598_2022_15003_MOESM1_ESM.tiff]

A)

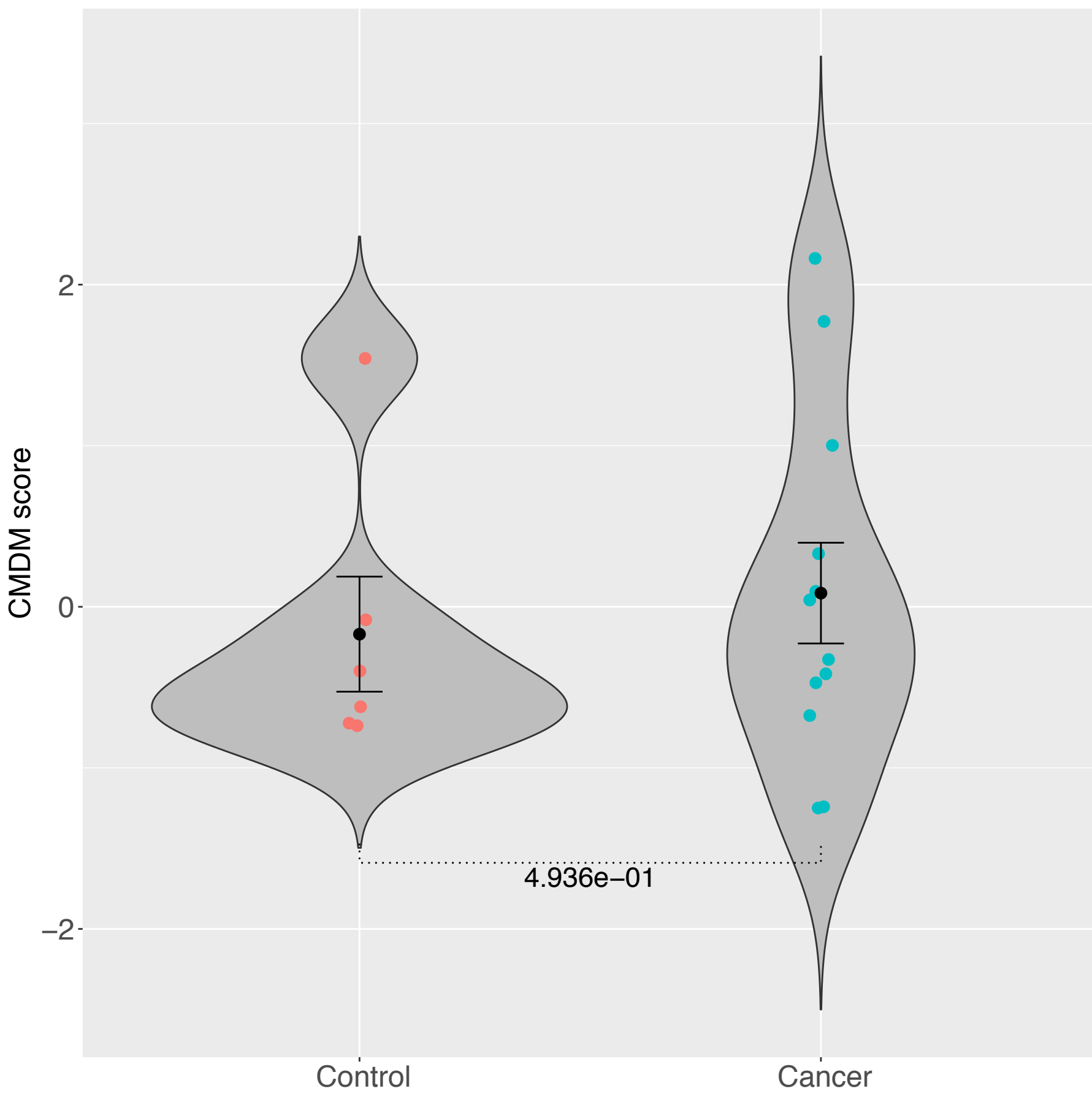

B)

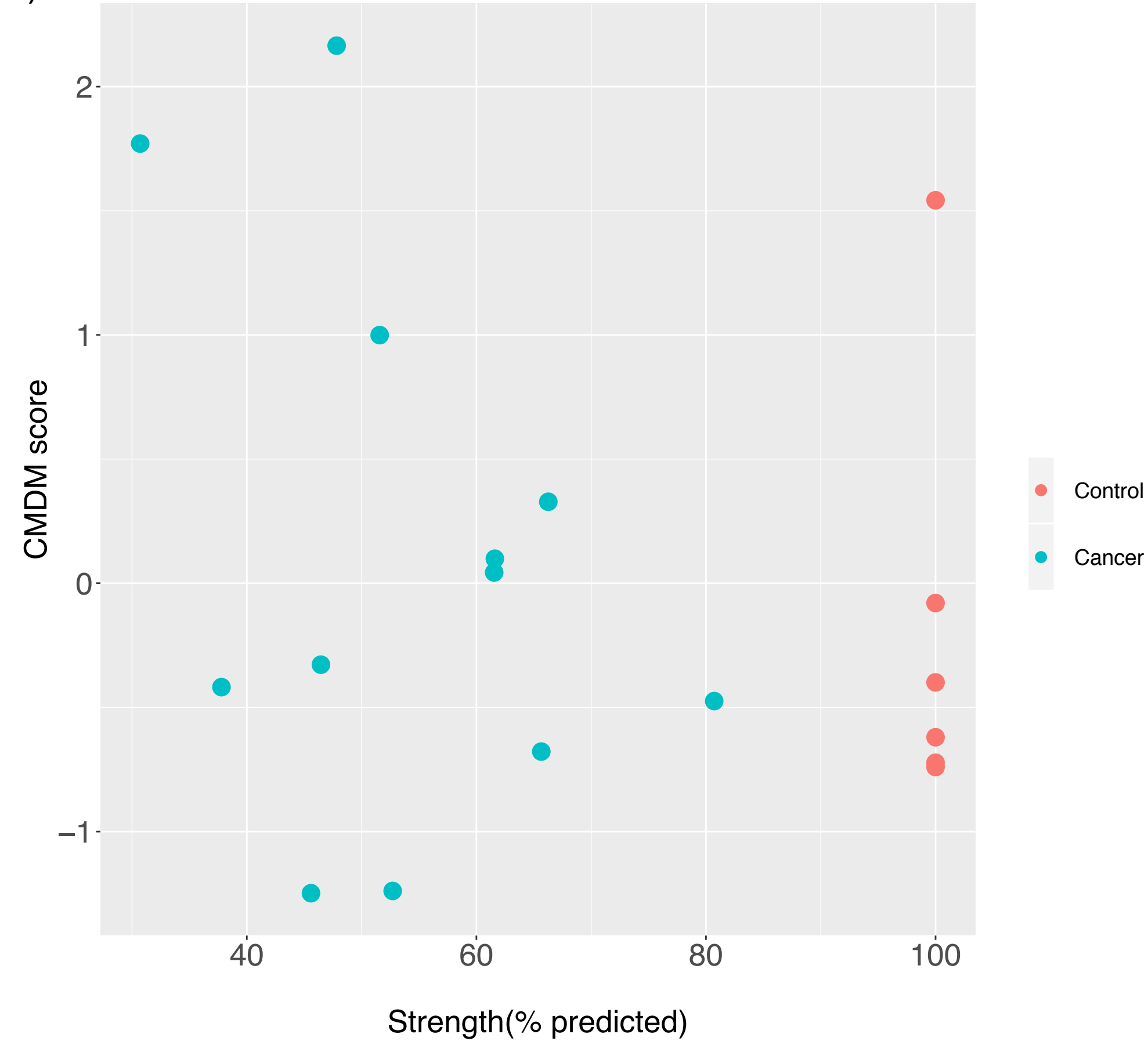

Supplement: Supplementary file 4 — Supplementary Figure 2. [file 41598_2022_15003_MOESM4_ESM.pdf]

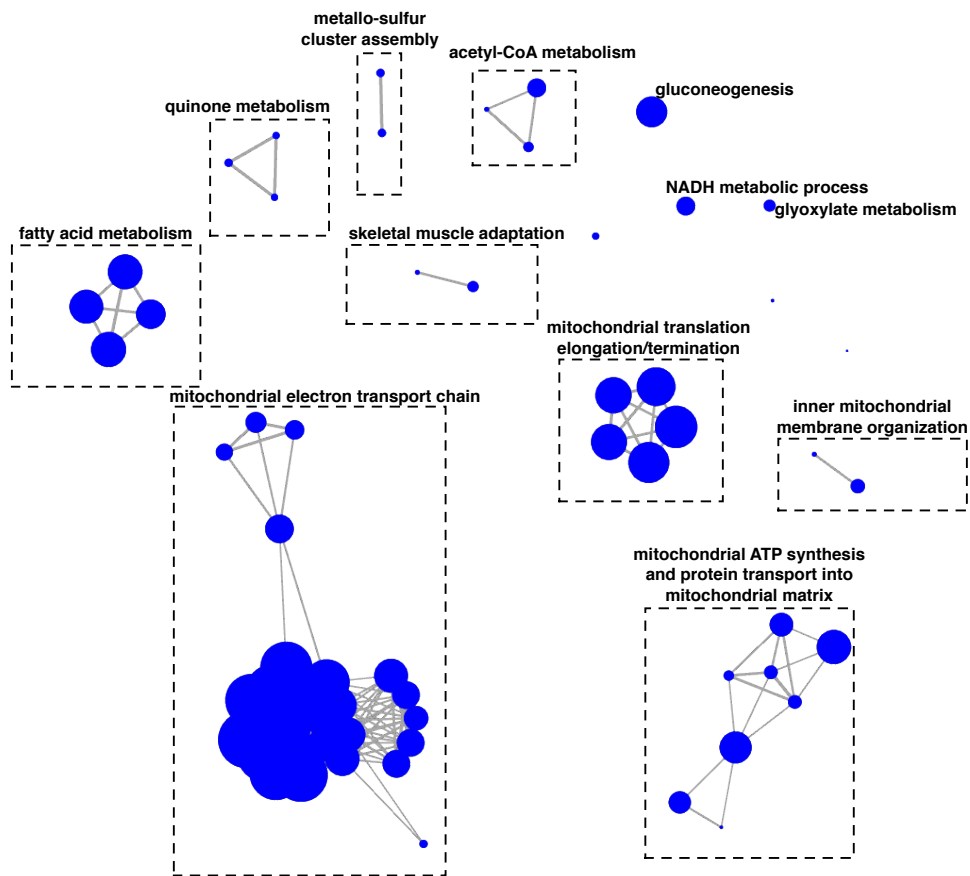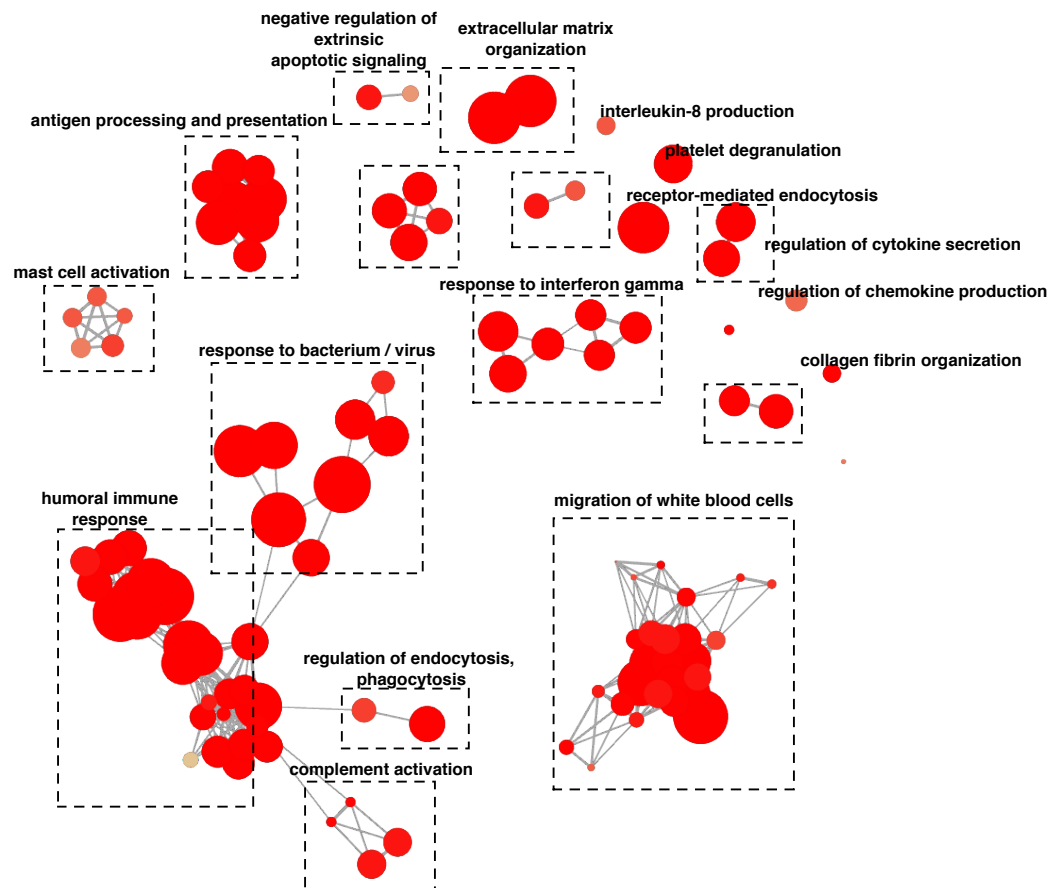

Supplement: Supplementary file 5 — Supplementary Figure 3. [file 41598_2022_15003_MOESM5_ESM.pdf]

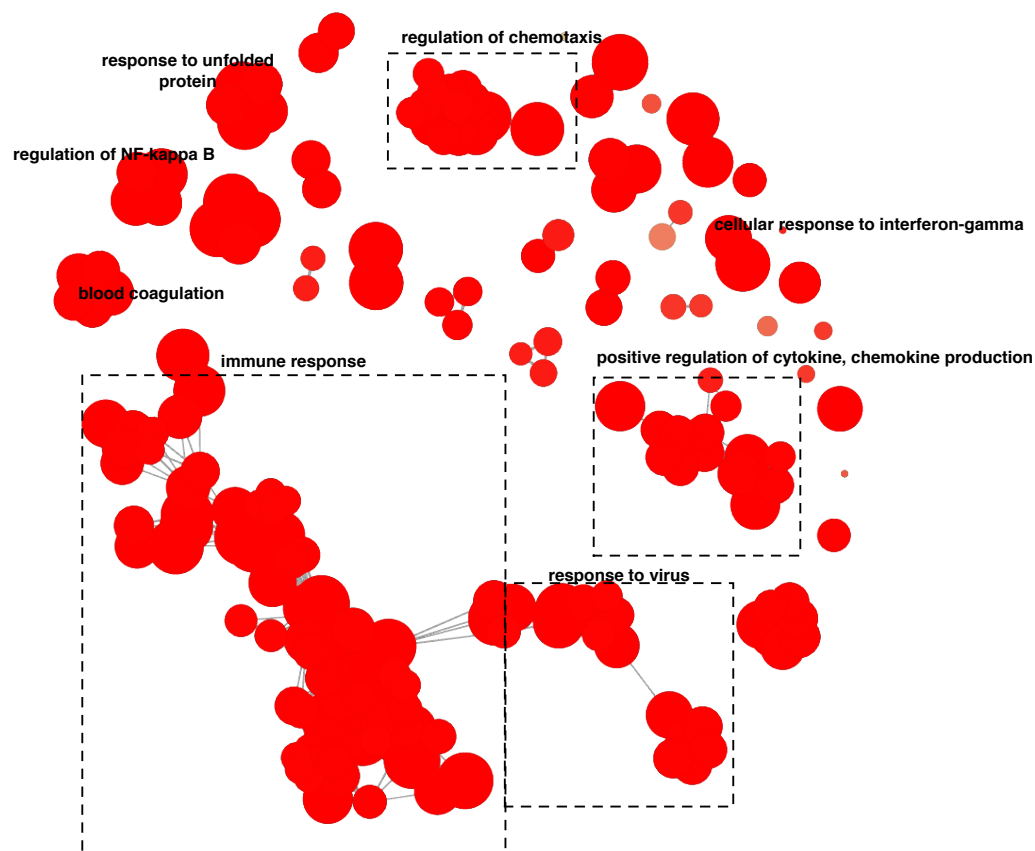

skeletal muscle contraction, filament sliding

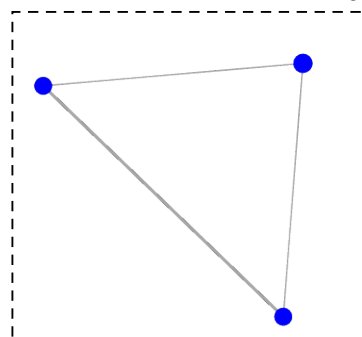

mitochondrial respiratory chain assembly

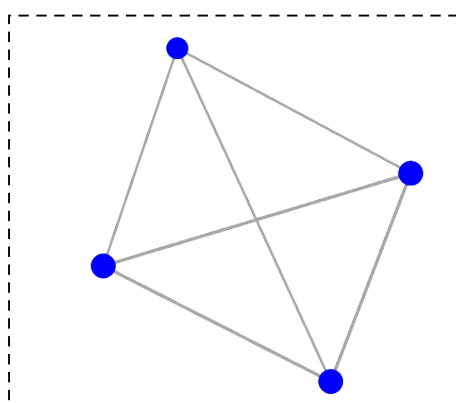

Supplement: Supplementary file 7 — Supplementary Figure 4B. [file 41598_2022_15003_MOESM7_ESM.pdf]

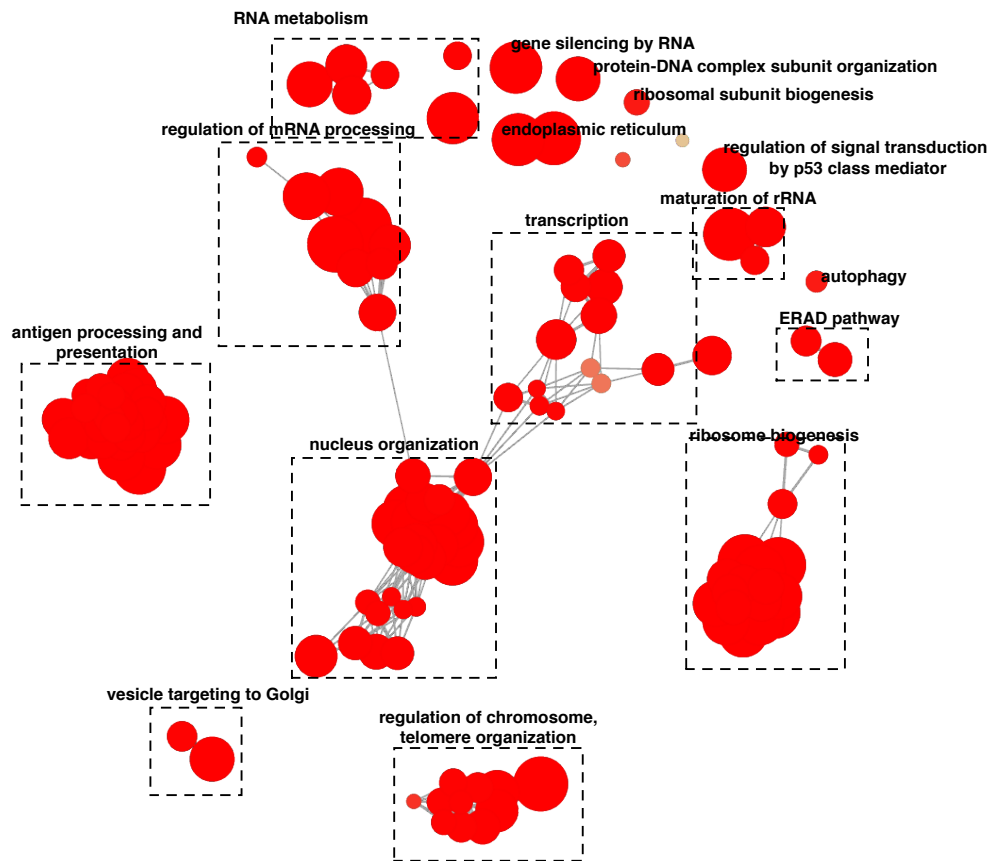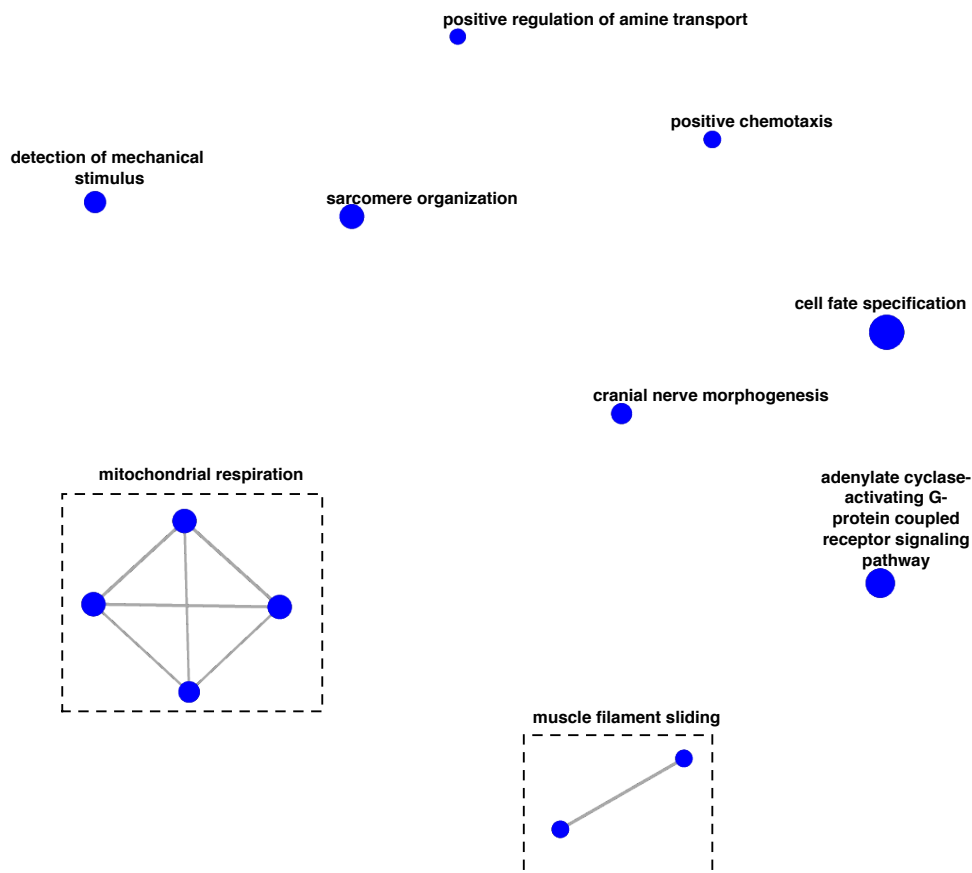

Supplement: Supplementary file 9 — Supplementary Figure 4D. [file 41598_2022_15003_MOESM9_ESM.pdf]

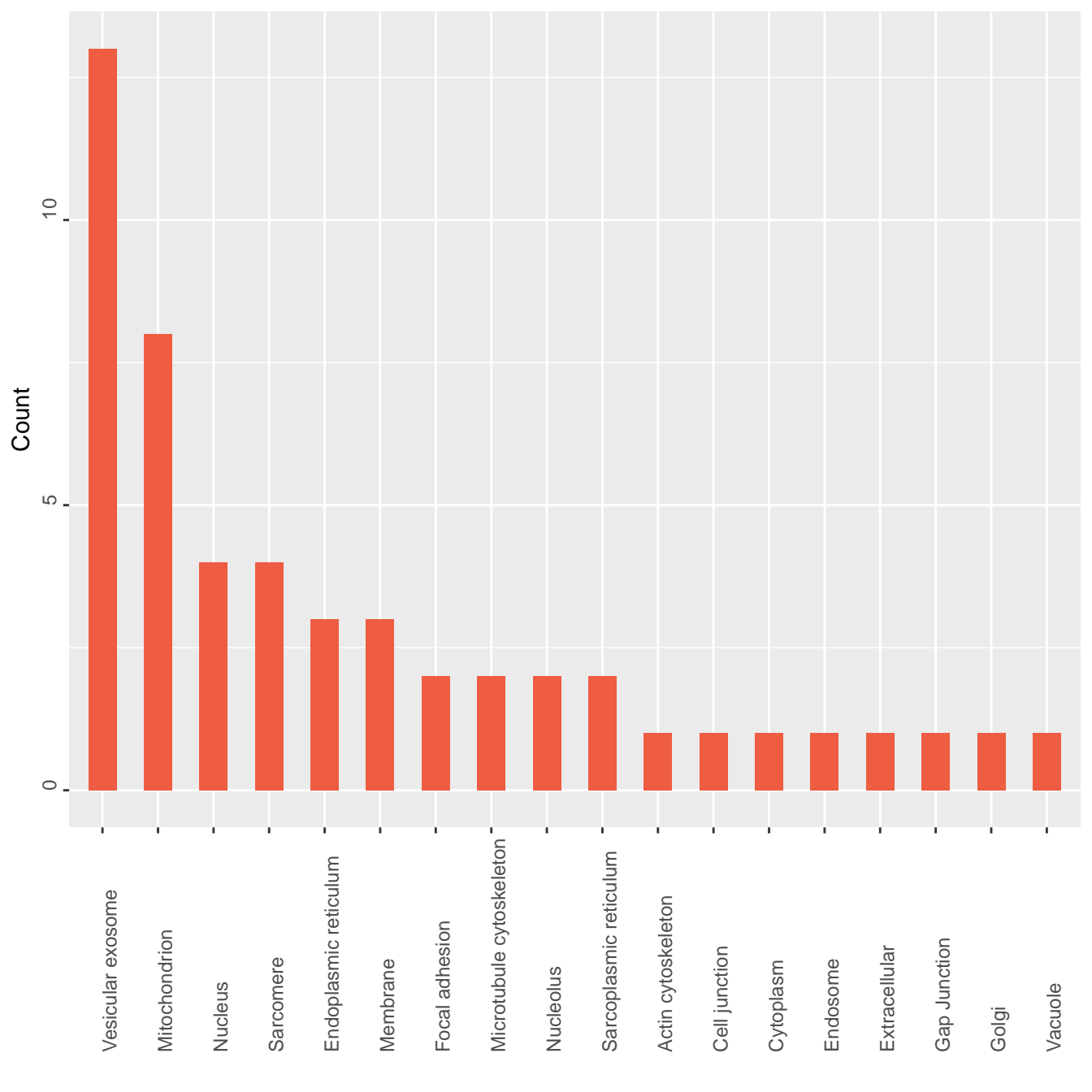

Supplement: Supplementary file 11 — Supplementary Figure 5. [file 41598_2022_15003_MOESM11_ESM.pdf]
