# Supplementary material for: Comprehensive multi-cohort transcriptional meta-analysis of muscle diseases identifies a signature of disease severity
Source: Sci Rep. 2022 Jul 4;12:11260. doi: 10.1038/s41598-022-15003-1 (PMC9253003; doi:10.1038/s41598-022-15003-1)
Supplement: Supplementary file 2 — Supplementary Information 1. [file 41598_2022_15003_MOESM2_ESM.docx]

Study summaries

This section describes each dataset used in the analysis. To ensure an accurate description of the datasets, their description has been used verbatim from their corresponding publications (or from public repository if unpublished) whenever possible. Whenever relevant, subgroups that were split or removed from our meta-analysis are specifically described (and shown in italics). If a study made available clinical or pathologic data for the skeletal muscle samples profiled, this is specifically indicated (and shown underlined). For unpublished datasets we have listed the first contributor in the public repository.

**GSE13205**  Fredriksson *et al* [1] profiled muscle biopsies obtained from the lateral portion of the *vastus lateralis* muscle, 10–20 cm above the knee in seventeen patients with sepsis admitted to the general Intensive Care Unit (ICU) at Karolinska University Hospital. Patients younger than 18 years of age, patients with severe liver failure, undergoing dialysis, and patients with impaired coagulation were excluded from the study. Ten patients undergoing elective surgery were included as a control group.

**GSE53702** Langhans *et al* [2] profiled biopsy specimens from *vastus lateralis* muscle in seven ICU patients. Three of the seven patients were diagnosed with critical illness myopathy (a subtype of ICU acquired weakness [ICUAW]) using muscle membrane inexcitability after direct muscle stimulation at day 6 ICU admission. Six patients undergoing elective orthopedic surgery without neuromuscular disorders were used as controls.

**GSE15090** Arashiro *et al* [3] profiled muscle biopsies to compared the gene-expression profiles from affected facioscapulohumeral muscular dystrophy (FSHD) individuals, asymptomatic carriers, and normal controls. Biopsies were taken from related members (affected, asymptomatic carrier, and normal control) belonging to 5 unrelated families. Muscle biopsies were taken from the biceps in 3 families and from the deltoid in the remaining 2 families, because the clinically affected patients had a severe atrophy. *Asymptomatic carriers were removed from our meta-analysis.*

**GSE18715** Voets (unpublished)

https://www.ncbi.nlm.nih.gov/geo/query/acc.cgi?acc=GSE18715 Skeletal muscle gene expression profiles of six patients with mutations in the catalytic DNA polymerase gamma (POLG1) gene (resulting in the accumulation of mtDNA mutations) were compared with twelve controls. Patient and control subject from three age categories (<10 yrs; 11-49 yrs; >50 yrs) were selected.

|  |  |
| --- | --- |

**GSE36398** Rahimov *et al* [4] aimed to better understand the pathophysiology of FSHD and develop mRNA-based biomarkers of affected muscles by profiling the *biceps*, which typically shows an early and severe disease involvement and *deltoid*, which are relatively uninvolved in patients with FSHD compared to controls biopsies of *biceps* and *deltoids*, respectively. Microarray samples were generated in 5 batches. *We have designated* *batches 1-4 as* ***GSE36398a****, and* ***GSE36398b*** *batch 5. These batches were separated based on principal components analysis.* *We have excluded biopsies obtained from the deltoids as these are relatively spared in FSHD.*

**GSE37084** Perfetti *et al* [5] profiled muscle biopsies from *biceps brachii* from ten patients with myotonic dystrophy type (DM2) where clinical diagnosis of DM2 was based upon the criteria set by the International Consortium for Myotonic Dystrophies [6]. Ten control biopsies were from subjects admitted with suspected neuromuscular disorder of undetermined nature. Control biopsies did not show overt signs of muscle pathology upon on histological and immunohistochemical examination. All muscle biopsies were processed by the same pathology team and each was analyzed by two expert pathologists. The aim of the study was the identification of new aberrant alternative splicing events in DM2 patients.

**GSE26852** Tasca *et al* [7] profiled muscle biopsies from FSHD and inflammatory myopathies (IM) as described below:

***FSHD cohort***: muscle biopsies from various peripheral muscle (*biceps femoris, paravertebral, quadriceps, biceps femoris*) in twelve FSHD patients and four 4 dysferlinopathies (limb-girdle muscular dystrophy type 2A; LGMD2B) (age range 28–35). Unrelated, genetically confirmed (D4Z4 EcoRI fragment ,40Kb) FSHD patients who had undergone lower limb muscle Magnetic Resonance Imaging (MRI) were considered as candidates for the study. Patients who met the inclusion criteria (i.e. i) having at least one muscle showing hyperintensity on T2-short tau inversion recovery (T2-STIR) sequences, or ii) having normal T1-Weighted and T2-STIR sequence signal on quadriceps muscle). Gene-expression was compared with seven normal controls (age range 18– 58).

***IM cohort***: muscle biopsies from seven immunopathologically characterized inflammatory myopathies (IM): 2 dermatomyositis (DM), 2 polymyositis (PM), 1 necrotizing myopathy and 2 IM with nonspecific histopathological features (age range 23–73). Gene-expression was compared with seven normal controls (age range 18– 58).

**GSE47968** Nakamori *et al* [8] profiled *quadriceps* muscle biopsies in eight patients with FSHD, eight patients with DM1, and seven patients with DM2 and eight healthy control samples. The objective of the study was to perform global analysis of alternative splicing in DM1 and DM2. Nonambulant individuals and patients with congenital or childhood onset of DM1 were excluded to eliminate confounding effects of muscle disuse or maldevelopment

**GSE42806** Screen *et al* [9] profiled skeletal muscle from distal muscles sites (*tibialis anterior, tibialis posterior, gastrocnemius lateralis, gastrocnemius medialis, soleus, extensor halluces longus, extensor digitorum longus, thigh posterior*) in seven patients with tibial muscular dystrophy (TMD) to analyze gene expression compared to five healthy controls. All patients were diagnosed based on DNA mutation testing. Range of ages 37–92.

**GSE38417** Dorsey (unpublished) https://www.ncbi.nlm.nih.gov/geo/query/acc.cgi?acc=GSE38417

Gene expression data is from RNA extracted from muscle biopsy samples taken from patients with Duchenne muscular dystrophy (DMD) or pathologically normal controls.

|  |
| --- |

**GSE38680** Palermo *et al* [10] profiled muscle biopsies from two cohorts of infantile-onset Pompe disease (Glycogen Storage Disease Type I) to identify transcriptional differences that may contribute to the disease phenotype. In the first cohort, biceps biopsies from 9 infantile-onset Pompe patients and 10 controls were compared. In a separate experiment, quadriceps biopsies from 11 Pompe patients at either 0, 12, or 52 weeks after the initiation of treatment with Myozyme were compared to quadriceps biopsies from 7 controls. *We have designated the latter experimental cohort (quadriceps biopsies) as* ***GSE38680a*** *and designated the biceps biopsies in the first cohort as* ***GSE38680b****.*

**GSE11681** Saenz *et al* [11] profiled skeletal muscle (*quadriceps, deltoid, or biceps brachialis*) in ten muscle biopsy samples of limb-girdle muscular dystrophy type 2A (LGMD2A) patients with in which molecular diagnosis was ascertained. Gene expression profiling was compared to ten normal muscle samples

**GSE12648** Eisenberg *et al* [12] profiled skeletal muscle specimens (deltoid, biceps, quadriceps, tibialis anterior, gluteus, paraspinal, triceps) from ten hereditary inclusion body myopathy (HIBM) patients carrying the M712T Persian Jewish founder mutation and presenting mild histological changes, compared with ten healthy matched control individuals. Only mildly HIBM-affected muscles biopsies were selected when possible in order to detect changes as primary as possible,

**GSE6011** Pescatori *et al* [13] profiled 23 quadriceps muscle biopsies from Duchenne muscular dystrophy (DMD) based on the absence of dystrophin immunoreactivity on *quadriceps* muscle sections. None of the participants at the time of biopsy was or had been under corticosteroid treatment. Control biopsies (*n* = 14) were from individuals who came to the hospital with a suspect metabolic disorder that was not confirmed by biochemical and histopathological studies. Control biopsies did not show signs of muscle pathology on histological and histochemical examination.

**GSE48280** Surez-Calvet *et al* [14] profiled skeletal muscle biopsies in five patients with dermatomyositis (DM), five with polymyositis (PM) and five with inclusion body myositis (IBM). The patients fulfilled established diagnostic criteria and did not received any ttreatment prior to biopsy. All DM and PM subjects were female, aged 25–71 years, while the IBM subjects were male (*n* = 3) and female (*n* = 2), aged 67–77. Samples from patients with a neoplasm or poor RNA yield were excluded. Five control muscle biopsies were obtained from subjects undergoing hip replacement surgery. Routine histological stains were normal.

**GSE1551** Greenberg *et al* [15] profiled skeletal muscle (*biceps, deltoids, quadriceps*) from 13 with patients with dermatomyositis (DM). Seven were treated with corticosteroids for a median duration of 12 days (range, 1–350 days) and 7 had never received immunosuppressive therapy. Biopsies were performed for clinical indications independent of the study. Expression was compared to 10 normal subjects patients without clinical or histological evidence of a neuromuscular disorder

**E-MEXP-2681** Bernasconi (unpublished)

https://www.ebi.ac.uk/arrayexpress/experiments/E-MEXP-2681/ Muscle biopsies were taken from 6 patients with dermatomyositis (DM), 4 with polymyositis (PM) and 5 not myopathic subjects as controls.

**GSE3307**. Bakay *et al* [16] examined disease-specific transcriptional profiles of normal skeletal muscle and 12 muscle disease groups to determine if these expression fingerprints provide either pathophysiology or diagnostic information for these diseases. We have organized the 12 muscle disease groups into appropriate cohorts as follows:

***i) ICUAW cohort:*** one disease group with critical care myopathy.

***ii) Inflammatory myopathy cohort***: one disease group with juvenile dermatomyositis

***iii) Congenital disease cohort***: seven groups of congenital diseases (Fascioscapulohumeral muscular dystrophy [FSHD], Emery Dreifuss muscular dystrophy [both X linked recessive emerin form; autosomal dominant Lamin A/C form], Becker muscular dystrophy, Duchenne muscular dystrophy, Calpain 3 (LGMD2A), dysferlin (LGMD2B), FKRP [fukutin related protein] ).

***iv) ALS cohort:*** One group of amyotrophic lateral sclerosis (ALS).

***v) upper motor neuron disease cohort***: one group with spastic paraplegia (SPG4, spastin)

*We separated i) – iv) into separate cohorts in our meta-analysis. Disease group v) was excluded as it contained only 4 samples and was not categorized among the other cohorts.*

**GSE45745** Barres *et al* [17] profiled *vastus lateralis* skeletal muscle samples obtained from 5 morbidly obese subjects immediately before and 6 months after Gastric Bypass (GB) surgery as well as from 6 lean healthy control subjects. *Samples obtained 6 months after GB surgery were excluded.*

**GSE21496** Reich *et al* [18] profiled left *vastus lateralis* of healthy, sedentary men (N = 7) at baseline and immediately following 48 hours of unloading via unilateral lower limb suspension and 24 hours of reloading. *Samples at baseline served as healthy controls, samples taken at 24 hours of reloading were excluded from our meta-analysis.*

**GSE5110** Urso *et al* [19] profiled biopsies taken from the *vastus lateralis* muscle of five men (20.4 +/- 0.5 yr) before and after 48-h immobilization.

**GSE24215** Alibegovic *et al* [20] profiled skeletal muscle biopsies of *vastus lateralis* in ten young healthy Caucasian men (24–27 yr old) pre-inactivity and after 10 day bed rest challenge. These subjects were admitted to the Steno Diabetes Center for 10 days and were not permitted to deviate from a half-recumbent position during this period. Toilet visits, limited to a total of 15 min/day, were allowed. Study subjects were allowed to use a laptop computer, watch television, and read in the bed. Skeletal muscle samples were collected in both the basal and insulin-stimulated state before and after bed rest and in the basal state after 4 weeks of retraining. *We excluded insulin-stimulated samples as well as samples taken after retraining from our meta-analysis.*

**GSE104999** Rullman *et al* [21] profiled skeletal muscle biopsies of *vastus lateralis* in 14 healthy male subjects at baseline and at 21 days bedrest. This was a substudy of the PlanHab study which also included hypoxic bedrest and hypoxic ambulation (not included in this dataset). Throughout both bedrest interventions, each subject remained in a horizontal position at all times and was allowed one pillow for the head and to occasionally lean on an elbow while eating or being transferred to a gurney. Muscular exercise was prohibited.

**GSE474** Park *et al* [22] profiled skeletal muscle samples obtained from the *rectus abdominus* during abdominal surgery for eight lean women (BMI 25 kg/m2), eight morbidly obese women (BMI 40 kg/m2), and eight obese patients (BMI 25-40 kg/m^2^). This study aimed to identify the mRNA of proteins involved in fat oxidation that may be reduced in obese and morbidly obese individuals.  *Obese patients were excluded in our meta-analysis.*

**GSE27536** Turan *et al* [23] profiled skeletal muscle biopsies from the vastus lateralis of 15 patients with stable chronic obstructive pulmonary disorder (COPD) and 12 age-matched healthy sedentary subjects before and after 8 weeks of a supervised endurance exercise program. Nine COPD patients had normal fat free mass index (FFMI, 21Kg/m2) and 6 COPD patients had low FFMI (16Kg/m2).

**GSE1786** Radom-Aizik *et al* [24] profiled skeletal muscle biopsies from vastus lateral from six COPD patients and five sedentary age-matched healthy men, before and after 3 months of exercise training.

**E-MTAB-3671** Kreiner *et al* [25] profiled skeletal muscle biopsies from trapezius in nine glucocorticoid-naive patients with newly diagnosed, untreated polymyalgia rheumatica (PMR) and 10 matched (age, sex, and BMI) non-PMR control subjects before and after treatment with 14 days prednisolone (20mg/day) in a comprehensive clinical experimental research program. In all patients, the trapezius muscle exhibited the symptoms characteristic of PMR, i.e. aching, tenderness and stiffness. Controlled chronic comorbidities were accepted in both groups.

**GSE78929** Walsh *et al* [26] profiled skeletal muscle biopsies from *vastus lateralis* in patients with ICUAW Day 7 (n=14) and Month 6 (n=10) post-ICU discharge and compared with 8 healthy control subjects obtained from previously banked specimens collected from consenting individuals. Clinical variables assessed in the cohort included the motor subscore of the Functional Independence Measure (FIM), global muscle strength measured by MRC sum score (MRCSS) and quadriceps cross sectional area (CSA), expressed as a percentage of published age and sex matched norms.

**GSE13608** Bachinski *et al* [27] profiled skeletal muscle biopsies from DM1, DM2, Becker muscular dystrophy (BMD), Duchenne muscular dystrophy (DMD), tibial muscular dystrophy (TMD), and myotonia congenita—autosomal dominant (MC-AD), DM-like, and normal individuals (both adult and fetal). *The 3 fetal healthy control samples were removed from the meta-analysis.*

**GSE109178** Dadgar *et al* [28] profiled skeletal muscle biopsies from 6 normal controls, 17 DMD (absence of dystrophin), 11 BMD (present but abnormal dystrophin), 7 LGMD2I (FKRP deficiency, a glycosylation defect), and 8 LGMD2B (DYSF). Patients had a broad range of ages, clinical severity of their disease, and histopathological findings, although all neuromuscular disease patients showed evidence of a dystrophic process (degeneration/regeneration of muscle fibers). The study sought to determine the mechanisms underlying failure of muscle regeneration that is observed in dystrophic muscle through hypothesis generation using muscle profiling data. The amount of fibrotic replacement (fibrosis) was visually approximated by the same evaluator (E.P. Hoffman), and divided into normal, mild, moderate, or severe fibrosis categories.

**GSE10760** Osborne *et al* [29] profiled skeletal muscle in vastus lateralis from 19 patients with FSHD compared to thirty healthy individuals profiled before and after antibody enhancement. The objective of the study was to identify pathways that are abnormally regulated early in the FSHD disease process

**GSE3112** Greenberg *et al* [30] profiled muscle biopsy specimens from 23 patients with inclusion body myositis (IBM), six with polymyositis (PM), and 11 controls without neuromuscular disease.

**GSE39454** Zhu *et al* [31] profiled various muscle groups (biceps, quadriceps, deltoid) from patients with inflammatory myopathies (5 necrotizing myopathy [NM], 8 DM, 8 PM and 10 IBM) compared to 5 normal controls. Normal controls were not suspected clinically to have neuromuscular disease; had normal muscle strength by examination; and showed normal serum CK levels. The objective of the study was to develop gene signatures to characterize myositis patients at the molecular level.

**GSE14901** Abadi *et al* [32] profiled skeletal muscle biopsies from vastus lateralis from recreationally active, non-smoking, healthy men (N=12) and women (N = 12) before, after 48 hours and 14 days of immobilization. Subjects had a randomly assigned leg immobilized using a knee brace and were provided with walking crutches such that there was no weight bearing on the immobilized leg. The purpose of this study was to examine changes in global gene transcription during immobilization-induced muscle atrophy in humans. Muscle strength testing was conducted at each session using a dynamometer and magnetic resonance imaging (MRI) was used to determine the cross-sectional area (CSA) of the vastus muscles.

**GSE45462** Chen *et al* [33] profiled medial gastrocnemius in 24 subjects (13 men, 11 women; mean age 26.7 ± 8.3 years) with an injury to the lower leg (closed malleolus fracture) treated conservatively with 6 weeks of cast immobilization and following immobilization each subject completed 6 weeks structured rehabilitation program focusing on progressive resistance training of the ankle plantar flexor muscles. Four longitudinal muscle biopsies were taken at the following time points: before (pre-rehab; post-immobilization), after 3 weeks of rehabilitation (early transcriptional changes) and immediately after 6 weeks of rehabilitation (chronic transcriptional changes). An additional muscle biopsy is taken at 4 months post-immobilization from the uninvolved (contralateral) medial gastrocnemius, which serves as a control sample. *For our meta-analysis we included the before (pre-rehab) and uninvolved gastrocnemius control sample and excluded the 3 weeks mid-rehab and 6 weeks post rehab.*

**GSE34111** Gallagher *et al* [34] profiled quadriceps biopsies in twelve patients with upper gastrointestinal cancer pre-resection (weight-loss 7%) and median 8 month post- resection follow-up (range, 5–12 months) Post-resection patients were disease-free and weight-stable for previous 2 months. Six healthy controls recruited from the community underwent single quadriceps biopsy. Maximum voluntary isometric quadriceps strength was measured using an established method. Data were normalized to body mass (N kg 1).

**GSE100281** Willis-Owen *et al* [35] profiled quadriceps biopsies in 79 patients with COPD (4 patients with mild, 24 patients with moderate, 32 with GOLD severe and 19 with very severe COPD) and 16 healthy age- and gender-matched controls.

**E-MEXP-3260** Pradat *et al* [36] profiled skeletal muscle in the middle pertion of the deltoid muscle of 9 patients with probable or definite ALS. Normal control deltoid muscle samples were taken from 10 subjects without any significant neurological history, who underwent a shoulder orthopedic surgery. All patients had sporadic ALS, and presented with symptoms of limb onset. They underwent a complete needle electromyography (EMG) investigation, as performed in the routine work-up of patients with ALS. EMG measures were obtained from the anterior portion of the deltoid muscle. The study was designed to identify gene expression changes in skeletal muscle that could reliably define the degree of disease severity. Patients were classified prior to biopsy based on EMG measures and the manual muscle testing of shoulder abductors (scored from 0 to 5, with 0 representing total paralysis and 5 normal strength, according to the Medical Research Council score).

**GSE31243** Smith *et al* [37] profiled skeletal muscle biopsies from both the gracilis and semitendinosus obtained from 10 patients with cerebral palsy undergoing medial hamstring lengthening surgery. The control group was 10 patients undergoing ACL reconstruction with hamstring autograft. This study was designed to gain further understanding of the skeletal muscle response to cerebral palsy using microarrays and correlating the transcriptional data with functional measures

REFERENCES

1. Fredriksson K, Tjader I, Keller P, Petrovic N, Ahlman B, Scheele C, Wernerman J, Timmons JA, Rooyackers O: **Dysregulation of mitochondrial dynamics and the muscle transcriptome in ICU patients suffering from sepsis induced multiple organ failure**. *PLoS One* 2008, **3**(11):e3686.

2. Langhans C, Weber-Carstens S, Schmidt F, Hamati J, Kny M, Zhu X, Wollersheim T, Koch S, Krebs M, Schulz H *et al*: **Inflammation-induced acute phase response in skeletal muscle and critical illness myopathy**. *PLoS One* 2014, **9**(3):e92048.

3. Arashiro P, Eisenberg I, Kho AT, Cerqueira AM, Canovas M, Silva HC, Pavanello RC, Verjovski-Almeida S, Kunkel LM, Zatz M: **Transcriptional regulation differs in affected facioscapulohumeral muscular dystrophy patients compared to asymptomatic related carriers**. *Proc Natl Acad Sci U S A* 2009, **106**(15):6220-6225.

4. Rahimov F, King OD, Leung DG, Bibat GM, Emerson CP, Jr., Kunkel LM, Wagner KR: **Transcriptional profiling in facioscapulohumeral muscular dystrophy to identify candidate biomarkers**. *Proc Natl Acad Sci U S A* 2012, **109**(40):16234-16239.

5. Perfetti A, Greco S, Fasanaro P, Bugiardini E, Cardani R, Garcia-Manteiga JM, Riba M, Cittaro D, Stupka E, Meola G *et al*: **Genome wide identification of aberrant alternative splicing events in myotonic dystrophy type 2**. *PLoS One* 2014, **9**(4):e93983.

6. Udd B, Meola G, Krahe R, Wansink DG, Bassez G, Kress W, Schoser B, Moxley R: **Myotonic dystrophy type 2 (DM2) and related disorders report of the 180th ENMC workshop including guidelines on diagnostics and management 3-5 December 2010, Naarden, The Netherlands**. *Neuromuscul Disord* 2011, **21**(6):443-450.

7. Tasca G, Pescatori M, Monforte M, Mirabella M, Iannaccone E, Frusciante R, Cubeddu T, Laschena F, Ottaviani P, Ricci E: **Different molecular signatures in magnetic resonance imaging-staged facioscapulohumeral muscular dystrophy muscles**. *PLoS One* 2012, **7**(6):e38779.

8. Nakamori M, Sobczak K, Puwanant A, Welle S, Eichinger K, Pandya S, Dekdebrun J, Heatwole CR, McDermott MP, Chen T *et al*: **Splicing biomarkers of disease severity in myotonic dystrophy**. *Ann Neurol* 2013, **74**(6):862-872.

9. Screen M, Raheem O, Holmlund-Hampf J, Jonson PH, Huovinen S, Hackman P, Udd B: **Gene expression profiling in tibial muscular dystrophy reveals unfolded protein response and altered autophagy**. *PLoS One* 2014, **9**(3):e90819.

10. Palermo AT, Palmer RE, So KS, Oba-Shinjo SM, Zhang M, Richards B, Madhiwalla ST, Finn PF, Hasegawa A, Ciociola KM *et al*: **Transcriptional response to GAA deficiency (Pompe disease) in infantile-onset patients**. *Mol Genet Metab* 2012, **106**(3):287-300.

11. Saenz A, Azpitarte M, Armananzas R, Leturcq F, Alzualde A, Inza I, Garcia-Bragado F, De la Herran G, Corcuera J, Cabello A *et al*: **Gene expression profiling in limb-girdle muscular dystrophy 2A**. *PLoS One* 2008, **3**(11):e3750.

12. Eisenberg I, Novershtern N, Itzhaki Z, Becker-Cohen M, Sadeh M, Willems PH, Friedman N, Koopman WJ, Mitrani-Rosenbaum S: **Mitochondrial processes are impaired in hereditary inclusion body myopathy**. *Hum Mol Genet* 2008, **17**(23):3663-3674.

13. Pescatori M, Broccolini A, Minetti C, Bertini E, Bruno C, D'Amico A, Bernardini C, Mirabella M, Silvestri G, Giglio V *et al*: **Gene expression profiling in the early phases of DMD: a constant molecular signature characterizes DMD muscle from early postnatal life throughout disease progression**. *FASEB J* 2007, **21**(4):1210-1226.

14. Suarez-Calvet X, Gallardo E, Nogales-Gadea G, Querol L, Navas M, Diaz-Manera J, Rojas-Garcia R, Illa I: **Altered RIG-I/DDX58-mediated innate immunity in dermatomyositis**. *J Pathol* 2014, **233**(3):258-268.

15. Greenberg SA, Pinkus JL, Pinkus GS, Burleson T, Sanoudou D, Tawil R, Barohn RJ, Saperstein DS, Briemberg HR, Ericsson M *et al*: **Interferon-alpha/beta-mediated innate immune mechanisms in dermatomyositis**. *Ann Neurol* 2005, **57**(5):664-678.

16. Bakay M, Wang Z, Melcon G, Schiltz L, Xuan J, Zhao P, Sartorelli V, Seo J, Pegoraro E, Angelini C *et al*: **Nuclear envelope dystrophies show a transcriptional fingerprint suggesting disruption of Rb-MyoD pathways in muscle regeneration**. *Brain* 2006, **129**(Pt 4):996-1013.

17. Barres R, Kirchner H, Rasmussen M, Yan J, Kantor FR, Krook A, Naslund E, Zierath JR: **Weight loss after gastric bypass surgery in human obesity remodels promoter methylation**. *Cell Rep* 2013, **3**(4):1020-1027.

18. Reich KA, Chen YW, Thompson PD, Hoffman EP, Clarkson PM: **Forty-eight hours of unloading and 24 h of reloading lead to changes in global gene expression patterns related to ubiquitination and oxidative stress in humans**. *J Appl Physiol (1985)* 2010, **109**(5):1404-1415.

19. Urso ML, Scrimgeour AG, Chen YW, Thompson PD, Clarkson PM: **Analysis of human skeletal muscle after 48 h immobilization reveals alterations in mRNA and protein for extracellular matrix components**. *J Appl Physiol (1985)* 2006, **101**(4):1136-1148.

20. Alibegovic AC, Sonne MP, Hojbjerre L, Bork-Jensen J, Jacobsen S, Nilsson E, Faerch K, Hiscock N, Mortensen B, Friedrichsen M *et al*: **Insulin resistance induced by physical inactivity is associated with multiple transcriptional changes in skeletal muscle in young men**. *Am J Physiol Endocrinol Metab* 2010, **299**(5):E752-763.

21. Rullman E, Mekjavic IB, Fischer H, Eiken O: **PlanHab (Planetary Habitat Simulation): the combined and separate effects of 21 days bed rest and hypoxic confinement on human skeletal muscle miRNA expression**. *Physiol Rep* 2016, **4**(8).

22. Park JJ, Berggren JR, Hulver MW, Houmard JA, Hoffman EP: **GRB14, GPD1, and GDF8 as potential network collaborators in weight loss-induced improvements in insulin action in human skeletal muscle**. *Physiol Genomics* 2006, **27**(2):114-121.

23. Turan N, Kalko S, Stincone A, Clarke K, Sabah A, Howlett K, Curnow SJ, Rodriguez DA, Cascante M, O'Neill L *et al*: **A systems biology approach identifies molecular networks defining skeletal muscle abnormalities in chronic obstructive pulmonary disease**. *PLoS Comput Biol* 2011, **7**(9):e1002129.

24. Radom-Aizik S, Kaminski N, Hayek S, Halkin H, Cooper DM, Ben-Dov I: **Effects of exercise training on quadriceps muscle gene expression in chronic obstructive pulmonary disease**. *J Appl Physiol (1985)* 2007, **102**(5):1976-1984.

25. Kreiner FF, Borup R, Nielsen FC, Schjerling P, Galbo H: **Gene expression profiling in patients with polymyalgia rheumatica before and after symptom-abolishing glucocorticoid treatment**. *BMC Musculoskelet Disord* 2017, **18**(1):341.

26. Walsh CJ, Batt J, Herridge MS, Mathur S, Bader GD, Hu P, Dos Santos CC: **Transcriptomic analysis reveals abnormal muscle repair and remodeling in survivors of critical illness with sustained weakness**. *Sci Rep* 2016, **6**:29334.

27. Bachinski LL, Sirito M, Bohme M, Baggerly KA, Udd B, Krahe R: **Altered MEF2 isoforms in myotonic dystrophy and other neuromuscular disorders**. *Muscle Nerve* 2010, **42**(6):856-863.

28. Dadgar S, Wang Z, Johnston H, Kesari A, Nagaraju K, Chen YW, Hill DA, Partridge TA, Giri M, Freishtat RJ *et al*: **Asynchronous remodeling is a driver of failed regeneration in Duchenne muscular dystrophy**. *J Cell Biol* 2014, **207**(1):139-158.

29. Osborne RJ, Welle S, Venance SL, Thornton CA, Tawil R: **Expression profile of FSHD supports a link between retinal vasculopathy and muscular dystrophy**. *Neurology* 2007, **68**(8):569-577.

30. Greenberg SA, Bradshaw EM, Pinkus JL, Pinkus GS, Burleson T, Due B, Bregoli L, O'Connor KC, Amato AA: **Plasma cells in muscle in inclusion body myositis and polymyositis**. *Neurology* 2005, **65**(11):1782-1787.

31. Zhu W, Streicher K, Shen N, Higgs BW, Morehouse C, Greenlees L, Amato AA, Ranade K, Richman L, Fiorentino D *et al*: **Genomic signatures characterize leukocyte infiltration in myositis muscles**. *BMC Med Genomics* 2012, **5**:53.

32. Abadi A, Glover EI, Isfort RJ, Raha S, Safdar A, Yasuda N, Kaczor JJ, Melov S, Hubbard A, Qu X *et al*: **Limb immobilization induces a coordinate down-regulation of mitochondrial and other metabolic pathways in men and women**. *PLoS One* 2009, **4**(8):e6518.

33. Chen YW, Gregory C, Ye F, Harafuji N, Lott D, Lai SH, Mathur S, Scarborough M, Gibbs P, Baligand C *et al*: **Molecular signatures of differential responses to exercise trainings during rehabilitation**. *Biomed Genet Genom* 2017, **2**(1).

34. Gallagher IJ, Stephens NA, MacDonald AJ, Skipworth RJ, Husi H, Greig CA, Ross JA, Timmons JA, Fearon KC: **Suppression of skeletal muscle turnover in cancer cachexia: evidence from the transcriptome in sequential human muscle biopsies**. *Clin Cancer Res* 2012, **18**(10):2817-2827.

35. Willis-Owen SAG, Thompson A, Kemp PR, Polkey MI, Cookson W, Moffatt MF, Natanek SA: **COPD is accompanied by co-ordinated transcriptional perturbation in the quadriceps affecting the mitochondria and extracellular matrix**. *Sci Rep* 2018, **8**(1):12165.

36. Pradat PF, Dubourg O, de Tapia M, di Scala F, Dupuis L, Lenglet T, Bruneteau G, Salachas F, Lacomblez L, Corvol JC *et al*: **Muscle gene expression is a marker of amyotrophic lateral sclerosis severity**. *Neurodegener Dis* 2012, **9**(1):38-52.

37. Smith LR, Chambers HG, Subramaniam S, Lieber RL: **Transcriptional abnormalities of hamstring muscle contractures in children with cerebral palsy**. *PLoS One* 2012, **7**(8):e40686.
