# Supplementary material for: Comprehensive multi-cohort transcriptional meta-analysis of muscle diseases identifies a signature of disease severity
Source: Sci Rep. 2022 Jul 4;12:11260. doi: 10.1038/s41598-022-15003-1 (PMC9253003; doi:10.1038/s41598-022-15003-1)
Supplement: Supplementary file 3 — Supplementary Legends. [file 41598_2022_15003_MOESM3_ESM.docx]

**Figure Legends for Supplementary Figures for** Comprehensive multi-cohort transcriptional meta-analysis of muscle diseases identifies a signature of disease severity

CJ Walsh^1,2^, J Batt^1,2^, M.S. Herridge^4^, S. Mathur^5^, GD Bader^6^, P Hu^7^, P Khatri^8,9^, and CC. dos Santos^1,3^ Submitted on behalf of the MEND-ICU Group and Canadian Critical Care Translational Biology Group

1. Keenan Research Center for Biomedical Science, Saint Michael’s Hospital, Toronto, Ontario, Canada.

2. Institute of Medical Sciences and Department of Medicine, University of Toronto, Toronto, Ontario, Canada.

3. Interdepartmental Division of Critical Care, University of Toronto, Toronto, Ontario, Canada

4. University Health Network, Interdepartmental Division of Critical Care, University of Toronto, Toronto, Ontario, Canada

5. Department of Physical Therapy, University of Toronto, Toronto, Ontario, Canada

6. The Donnelly Center, University of Toronto, Toronto, Ontario, Canada

7. Department of Biochemistry and Medical Genetics, University of Manitoba, Winnipeg, Manitoba, Canada

8. Stanford Institute for Immunity, Transplantation and Infection (ITI), Stanford University School of Medicine, Stanford, CA, USA

9. Stanford Center for Biomedical Informatics Research (BMIR), Department of Medicine, Stanford University, Stanford, CA, USA

**Supplementary Figure 1**: Meta-analysis workflow diagram (see Methods section for details). GEO, Gene Expression Omnibus; FDR, False Discovery Rate; AUC, Area under the Curve.

**Supplementary Figure 2**: CMDM signature in cancer cachexia (GSE34111). Violin plots of A) CMDM z-scores (muscle disease severity score) for cancer cachexia vs controls, B) CMDM z-scores vs quadriceps strength (% predicted).

**Supplementary Figure 3**: Functional enrichment reveals common pathways in muscle disease. EnrichmentMap network for overlapping enriched Gene Ontology gene sets identified by GSEA. Each node represents a significantly enriched gene set (FDR q-value < 0.05); gene sets containing larger number of genes are proportionally larger. Gene Sets downregulated in muscle disease compared to control shown in blue (top) and gene sets upregulated shown in red (bottom).

**Supplementary Figure 4A-D**: Functional enrichment reveals common pathways in A) congenital muscle disease B) inflammatory myopathies C) disuse and immobility D) ICU acquired weakness E) chronic systemic diseases. EnrichmentMap network for overlapping enriched Gene Ontology gene sets identified by GSEA. Each node represents a significantly enriched gene set (FDR q-value < 0.05); gene sets containing larger number of genes are proportionally larger. Gene Sets upregulated in muscle disease compared to control shown in red (top) and gene sets downregulated shown in blue (bottom).

**Supplementary Figure 5**: Bar graph of genes in the CMDM by subcellular localization.
