# Supplementary material for: Comprehensive multi-cohort transcriptional meta-analysis of muscle diseases identifies a signature of disease severity
Source: Sci Rep. 2022 Jul 4;12:11260. doi: 10.1038/s41598-022-15003-1 (PMC9253003; doi:10.1038/s41598-022-15003-1)
Supplement: Supplementary file 6 — Supplementary Figure 4A. [file 41598_2022_15003_MOESM6_ESM.pdf]

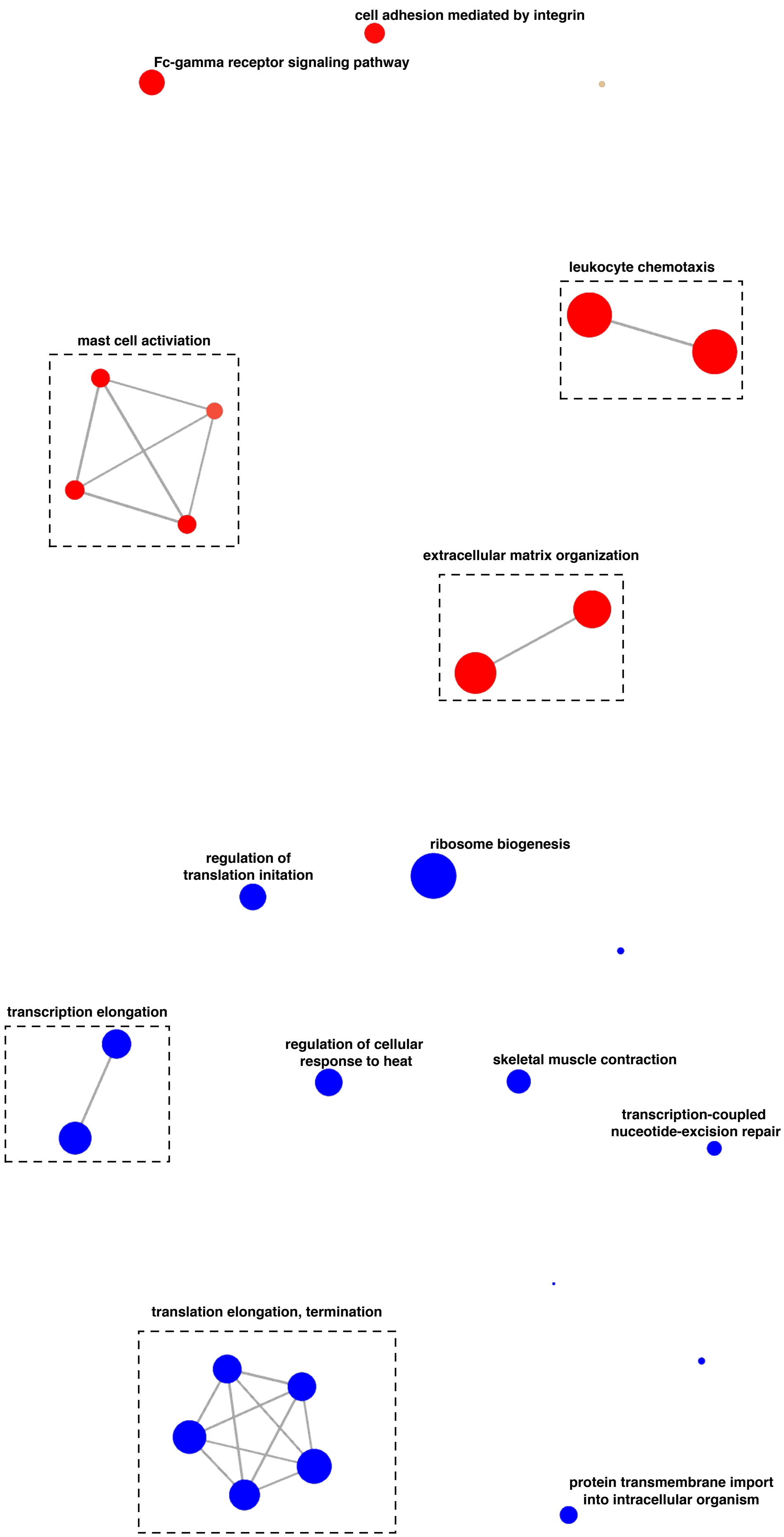
cell adhesion mediated by integrin
Fc-gamma receptor signaling pathway
leukocyte chemotaxis
mast cell activiation
extracellular matrix organization
ribosome biogenesis
regulation of
translation initation
transcription elongation
regulation of cellular
response to heat
skeletal muscle contraction
transcription-coupled
nuceotide-excision repair
translation elongation, termination
protein transmembrane import
into intracellular organism
