# Supplementary material for: Comprehensive multi-cohort transcriptional meta-analysis of muscle diseases identifies a signature of disease severity
Source: Sci Rep. 2022 Jul 4;12:11260. doi: 10.1038/s41598-022-15003-1 (PMC9253003; doi:10.1038/s41598-022-15003-1)
Supplement: Supplementary file 8 — Supplementary Figure 4C. [file 41598_2022_15003_MOESM8_ESM.pdf]

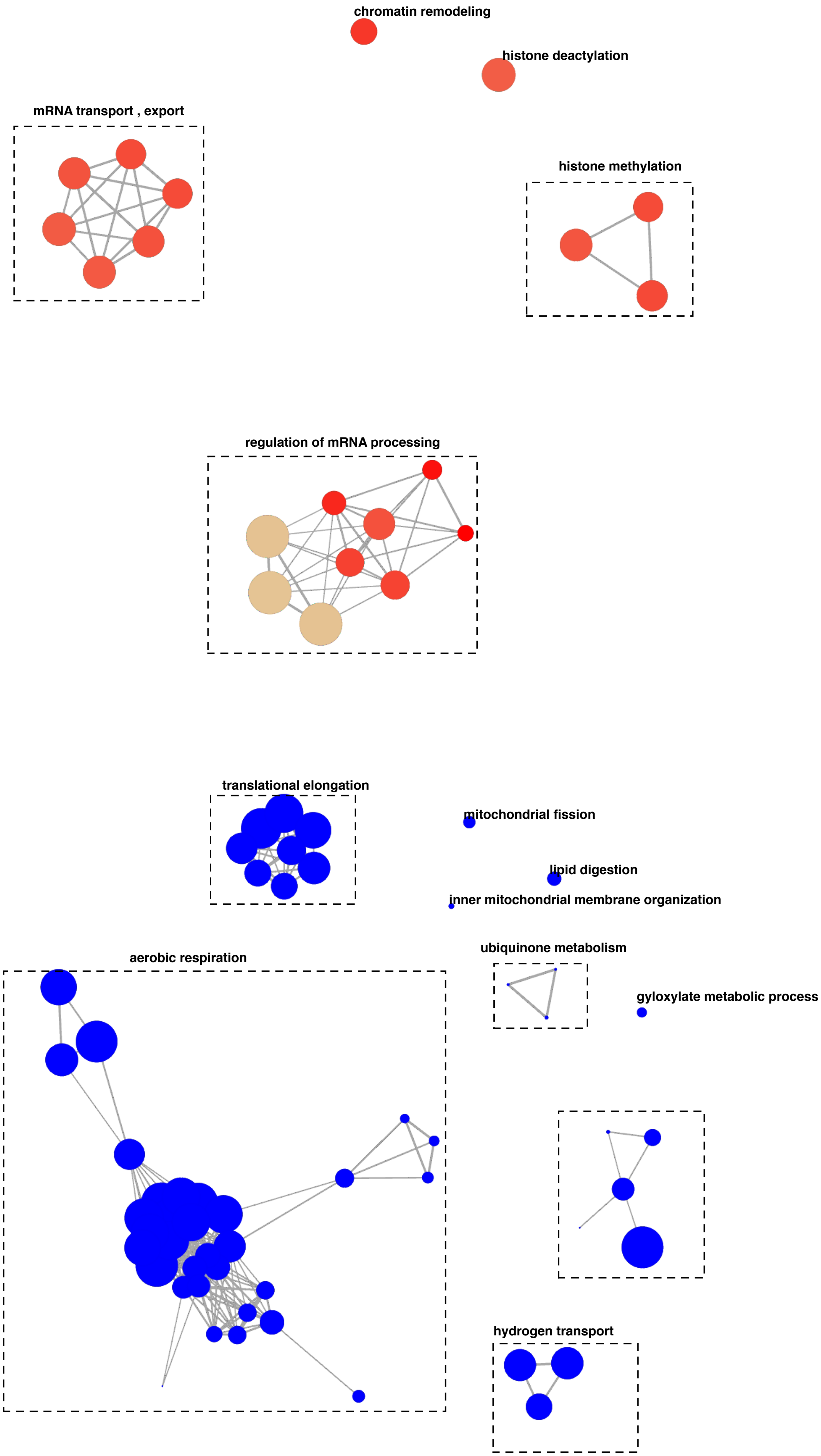

chromatin remodeling
histone deactylation
mRNA transport , export
histone methylation
regulation of mRNA processing
translational elongation
mitochondrial fission
lipid digestion
inner mitochondrial membrane organization
aerobic respiration
ubiquinone metabolism
gyloxylate metabolic process
hydrogen transport
