# Supplementary material for: Comprehensive multi-cohort transcriptional meta-analysis of muscle diseases identifies a signature of disease severity
Source: Sci Rep. 2022 Jul 4;12:11260. doi: 10.1038/s41598-022-15003-1 (PMC9253003; doi:10.1038/s41598-022-15003-1)
Supplement: Supplementary file 10 — Supplementary Figure 4E. [file 41598_2022_15003_MOESM10_ESM.pdf]

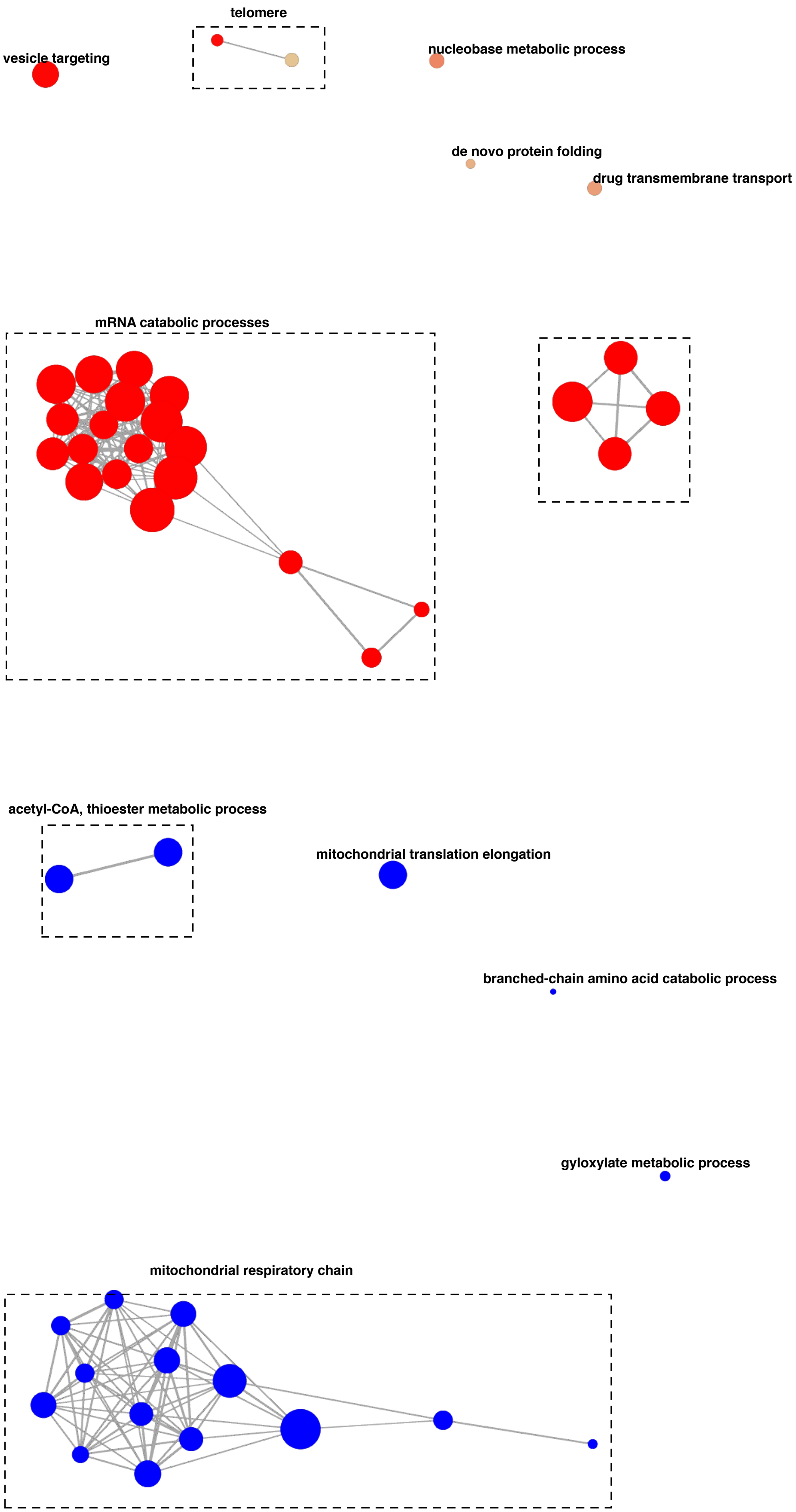

telomere
vesicle targeting
nucleobase metabolic process
de novo protein folding
drug transmembrane transport
mRNA catabolic processes
acetyl-CoA, thioester metabolic process
mitochondrial translation elongation
branched-chain amino acid catabolic process
gyloxylate metabolic process
mitochondrial respiratory chain
