# Supplementary material for: Comprehensive multi-cohort transcriptional meta-analysis of muscle diseases identifies a signature of disease severity
Source: Sci Rep. 2022 Jul 4;12:11260. doi: 10.1038/s41598-022-15003-1 (PMC9253003; doi:10.1038/s41598-022-15003-1)
Supplement: Supplementary file 12 — Supplementary Information 2. [file 41598_2022_15003_MOESM12_ESM.docx]

**Supplementary Methods for** Comprehensive multi-cohort transcriptional meta-analysis of muscle diseases identifies a signature of disease severity

CJ Walsh^1,2^, J Batt^1,2^, M.S. Herridge^4^, S. Mathur^5^, GD Bader^6^, P Hu^7^, P Khatri^8,9^, and CC. dos Santos^1,3^ Submitted on behalf of the MEND-ICU Group and Canadian Critical Care Translational Biology Group

1. Keenan Research Center for Biomedical Science, Saint Michael’s Hospital, Toronto, Ontario, Canada.

2. Institute of Medical Sciences and Department of Medicine, University of Toronto, Toronto, Ontario, Canada.

3. Interdepartmental Division of Critical Care, University of Toronto, Toronto, Ontario, Canada

4. University Health Network, Interdepartmental Division of Critical Care, University of Toronto, Toronto, Ontario, Canada

5. Department of Physical Therapy, University of Toronto, Toronto, Ontario, Canada

6. The Donnelly Center, University of Toronto, Toronto, Ontario, Canada

7. Department of Biochemistry and Medical Genetics, University of Manitoba, Winnipeg, Manitoba, Canada

8. Stanford Institute for Immunity, Transplantation and Infection (ITI), Stanford University School of Medicine, Stanford, CA, USA

9. Stanford Center for Biomedical Informatics Research (BMIR), Department of Medicine, Stanford University, Stanford, CA, USA

**Data collection and pre-processing**

Two public gene expression microarray repositories (ArrayExpress, NIH GEO) (search date: Aug 29, 2019) were searched for human muscle disease datasets using the search terms “skeletal muscle”, “myopathy”, “dystrophy”, “cachexia”, “immobility”, “bed rest”, “unloading”, “disuse”, “paralysis”, “spinal cord injury (SCI)”, “morbid obesity”, “critical illness”, “ICU”, “sepsis”, “myositis”, “dermatomyositis”, “polymositis”, “inclusion body myositis”, “chronic obstructive pulmonary disease (COPD)”, “cancer”, “amyotrophic lateral sclerosis (ALS)”, “hereditary spastic paraplegia (HSP)”, “primary lateral sclerosis (PLS)”, “progressive muscular atrophy (PMA)” , “spinal muscular atrophy (SMA)”, “motor neuron disease”, “mitochondrial”, “symptom”, “weakness”, “muscle mass”. We first identified data sets that satisfied the following criteria: (1) samples were from human peripheral muscle tissue, (2) data was originally acquired using a genome-wide gene expression microarray platform with probes representing > 10,000 genes, (3) the microarray platform had reasonably accessible and clear probe-to-gene mapping annotations and (4) there were >= 5 cases and >= 5 controls total for the relevant patient cohort in each data set and (5) the controls were taken from healthy muscle tissue. Samples that were taken after intervention (e.g. after leg casting or gastric bypass surgery) were excluded.

Some data sets included more than one disease category therefore we refer to each disease-specific group and its respective control group as a *patient cohort*. Two patient cohorts (GSE36398 and GSE38680) were each divided into two separate cohorts (designated by the suffix “a” and “b”) due to batch effects identified during the preliminary analysis. Datasets containing a subset of samples included in a large dataset were removed from further analysis to avoid redundant samples. We identified a total of 45 cohorts containing 1374 samples from 41 independent data sets that satisfied these criteria.

To ensure similar normalization methods, all microarray data were renormalized from raw data (when available) using standardized methods. Affymetrix arrays were normalized using GC robust multiarray average (gcRMA) on arrays with mismatch probes or RMA (R package “affy”). Gene detection (presence/absence calls) on Affymetrix HG-U133 series microarray data was performed using default parameters using R package *panp*). For all non-Affymetrix arrays, we downloaded data in non-normalized form, background corrected using the normal-exponential method, and then quantile normalized (R package *limma*) . All probe-to-gene mappings were derived from the most current SOFT files in GEO (downloaded on March 5, 2018). All microarray data were log-2 transformed and the expression of probes were summarized to the expression of genes within datasets using a fixed-effect inverse variance model [1]. A total of 24,572 genes were measured in at least one discovery and validation cohort. We restricted the gene lists to genes that were measured in a minimum of 80% of the discovery cohorts. For analysis of combined cohorts, a minimum of 50% of cohorts was used.

**Meta-analysis**

Multicohort meta-analysis of gene expression was performed combing gene effect sizes (Hedges’ g ) using a DerSimonian-Laird random-effects model using false discovery rate (FDR) via Benjamini-Hochberg method. We set significance threshold for differential expression at FDR less than 10% and effect size greater than 0.6. The choice of model and thresholds for gene selection were based on prior analysis of different meta-analysis models [2]. In order to ensure that our meta-analysis was not biased towards a specific muscle disease category we repeated our meta-analysis 5 times by removing data sets corresponding to one disease at a time (e.g. in the first iteration, ICU-acquired weakness data sets were removed, and the meta-analysis was completed on the combined remaining data sets). At each iteration, we identified significant DE genes (FDR ≤ 10%). Genes that were significant, irrespective of which subset of muscle disease category were selected for gene signature discovery.

**Derivation of Common Muscle Disease Module (CMDM) score**

Genes in the CMDM were separated according to whether their effect sizes were positive or negative (where “positive” means a positive effect size in muscle disease as compared to healthy controls, and “negative” means a negative effect size in muscle disease compared to healthy controls) and the geometric mean of the gene expression intensity for the up-regulated and down-regulated genes within the CMDM were calculated separately within each sample. The geometric mean of the CMDM was centered and standardized across all samples in a given experiment given a z-score. The difference between the up-regulated and the down regulated z-score in each sample is hereafter termed the CMDM score, used in this analysis. We calculated a CMDM score for each sample by subtracting the geometric mean expression of the 16 down-regulated genes from the 36 up-regulated genes.

We applied a greedy forward search using the MetaIntegrator R package [1] to identify a parsimonious gene signature maximized for diagnostic power. The greedy forward search is an algorithm that seeks to identify the set of genes that best distinguishes two groups (e.g. controls and diseases) by iteratively finding the best set of genes among the candidates. We set an arbitrary minimum threshold for performance of a mean area under the receive characteristic curve (AUROC) of 0.85 in the discovery data. We identified three gene sets with a total of 52 genes, termed the Common Muscle Disease Module (CMDM). The entire list of 52 genes was then pooled to make a single CMDM score. . The CMDM score was tested for diagnostic power using receiver operated characteristic (ROC) curves.

**Gene ontology functional analysis**

Gene Set Enrichment Analysis (GSEA) was used to identify enrichment of the genes differentially expressed (DE) in the muscle meta-analyses (without arbitrary thresholds for significance of DE) using pre-established curated gene sets in Gene Ontology (GO). To identify functional themes across all muscle disease categories from the discovery and validation meta-analysis, the complete list of genes with corresponding meta-effect sizes were input into GSEA PreRank, performing 100,000 permutations for assessment of GO term enrichment using FDR < 5% and normalized effect size > absolute(1.0) as threshold for significance (implemented in ClusterProfiler package). After removing redundant enriched GO terms using semantic similarity [3], the significantly enriched GO terms were visualized as a network based on their overlapping genes using EnrichentMap [4].

The individual disease meta-analysis gene lists generated using muscle disease category specific meta-analysis were also input into GSEA PreRank for assessment of GO term enrichment as described above.

**Subcellular localization analysis**

Subcellular localization information from two carefully curated sources (UniProt and Gene Ontology) was downloaded from CellWhere [5]. Subcellular localizations were assigned priority scores according to the location’s relevance to muscle physiology (‘Muscle flavor priority’). For genes having more than one subcellular annotation, the subcellular annotation having the highest muscle priority score was selected.

For each subcellular localization, Fisher’s exact test was performed to assess enrichment of CMDM genes in the subcellular localization using all of the genes in the discovery analysis as background.

References

1. Haynes WA, Vallania F, Liu C, Bongen E, Tomczak A, Andres-Terre M, Lofgren S, Tam A, Deisseroth CA, Li MD *et al*: **Empowering Multi-Cohort Gene Expression Analysis to Increase Reproducibility**. *Pac Symp Biocomput* 2017, **22**:144-153.

2. Sweeney TE, Haynes WA, Vallania F, Ioannidis JP, Khatri P: **Methods to increase reproducibility in differential gene expression via meta-analysis**. *Nucleic Acids Res* 2017, **45**(1):e1.

3. Wang JZ, Du Z, Payattakool R, Yu PS, Chen CF: **A new method to measure the semantic similarity of GO terms**. *Bioinformatics* 2007, **23**(10):1274-1281.

4. Merico D, Isserlin R, Stueker O, Emili A, Bader GD: **Enrichment map: a network-based method for gene-set enrichment visualization and interpretation**. *PLoS One* 2010, **5**(11):e13984.

5. Zhu L, Malatras A, Thorley M, Aghoghogbe I, Mer A, Duguez S, Butler-Browne G, Voit T, Duddy W: **CellWhere: graphical display of interaction networks organized on subcellular localizations**. *Nucleic Acids Res* 2015, **43**(W1):W571-575.
